# Antagonistic regulation of mRNA expression and splicing by CELF and MBNL proteins

**DOI:** 10.1101/gr.184390.114

**Published:** 2015-06

**Authors:** Eric T. Wang, Amanda J. Ward, Jennifer M. Cherone, Jimena Giudice, Thomas T. Wang, Daniel J. Treacy, Nicole J. Lambert, Peter Freese, Tanvi Saxena, Thomas A. Cooper, Christopher B. Burge

**Affiliations:** 1 Department of Biology, Massachusetts Institute of Technology, Cambridge, Massachusetts 02142, USA;; 2 Koch Institute for Integrative Cancer Research, Massachusetts Institute of Technology, Cambridge, Massachusetts 02142, USA;; 3 Department of Pathology and Immunology, Baylor College of Medicine, Houston, Texas 77030, USA;; 4 Department of Molecular and Cellular Biology, Baylor College of Medicine, Houston, Texas 77030, USA;; 5 Department of Molecular Physiology and Biophysics, Baylor College of Medicine, Houston, Texas 77030, USA

## Abstract

RNA binding proteins of the conserved CUGBP1, Elav-like factor (CELF) family contribute to heart and skeletal muscle development and are implicated in myotonic dystrophy (DM). To understand their genome-wide functions, we analyzed the transcriptome dynamics following induction of *CELF1* or *CELF2* in adult mouse heart and of *CELF1* in muscle by RNA-seq, complemented by crosslinking/immunoprecipitation-sequencing (CLIP-seq) analysis of mouse cells and tissues to distinguish direct from indirect regulatory targets. We identified hundreds of mRNAs bound in their 3′ UTRs by both CELF1 and the developmentally induced MBNL1 protein, a threefold greater overlap in target messages than expected, including messages involved in development and cell differentiation. The extent of 3′ UTR binding by CELF1 and MBNL1 predicted the degree of mRNA repression or stabilization, respectively, following *CELF1* induction. However, CELF1's RNA binding specificity in vitro was not detectably altered by coincubation with recombinant MBNL1. These findings support a model in which CELF and MBNL proteins bind independently to mRNAs but functionally compete to specify down-regulation or localization/stabilization, respectively, of hundreds of mRNA targets. Expression of many alternative 3′ UTR isoforms was altered following *CELF1* induction, with 3′ UTR binding associated with down-regulation of isoforms and genes. The splicing of hundreds of alternative exons was oppositely regulated by these proteins, confirming an additional layer of regulatory antagonism previously observed in a handful of cases. The regulatory relationships between CELFs and MBNLs in control of both mRNA abundance and splicing appear to have evolved to enhance developmental transitions in major classes of heart and muscle genes.

CELF RNA binding proteins (RBPs) play roles in early embryonic development, heart, and skeletal muscle functions. They are also thought to contribute to DM pathogenesis (Timchenko et al. 1996) and have been suggested to contribute to other diseases (Ladd 2013). The six family members present in mammals can be divided into two subfamilies: *CELF1-2*, which are expressed most highly in heart, skeletal muscle, and brain, and *CELF3-6*, which exhibit more restricted expression (Dasgupta and Ladd 2012). The CELF proteins contain two N-terminal RNA recognition motifs (RRMs) and one C-terminal RRM, with which they bind GU-rich RNAs, and a linker region termed the “divergent domain” that separates RRM2 and RRM3 and is involved in the regulation of alternative pre-mRNA splicing and mRNA decay (Han and Cooper 2005; Vlasova et al. 2008).

During normal development, CELF1 and CELF2 proteins are highly expressed in early embryonic stages and are then down-regulated more than 10-fold in skeletal muscle (Ladd et al. 2005) and the heart (Kalsotra et al. 2008) during post-natal development, remaining at low levels in adult tissues. This developmental down-regulation occurs through multiple mechanisms, including repression by microRNAs (miRNAs) and reductions in protein phosphorylation, which destabilizes the protein (Kalsotra et al. 2010, 2014). However, in DM type 1 (DM1), CELF1 protein levels increase in skeletal muscle and heart (Timchenko et al. 2004) as the protein is stabilized by PKC-mediated phosphorylation (Kuyumcu-Martinez et al. 2007).

The combination of increased CELF levels and MBNL sequestration by CUG repeat RNA is thought to be responsible for much of DM pathology by reversing the developmental changes in both proteins toward embryonic levels, shifting splicing of regulatory targets toward fetal isoforms (Philips et al. 1998; Ho et al. 2004; Lin et al. 2006; Kuyumcu-Martinez et al. 2007). Expression of *CELF1* in adult mice recapitulates a subset of the misregulated splicing events observed in DM1 skeletal muscle and heart (Kalsotra et al. 2008, 2010; Ward et al. 2010). Of 44 developmentally regulated alternative splicing events in heart development that were investigated, 24 were found to revert toward embryonic splicing levels in response to inducible expression of *CELF1* in the adult heart or in mice lacking *Mbnl1* (Kalsotra et al. 2008). A long-standing question has been whether CELF and MBNL splicing factors share regulatory targets and whether they synergize or antagonize. The half-dozen events known to be regulated by both CELF and MBNL proteins, including *H2afy* exon 6 and *Mbnl2* exon 8, are regulated antagonistically (Ho et al. 2005; Dhaenens et al. 2008; Kalsotra et al. 2008). However, in a study analyzing CELF motifs present near *Mbnl*-responsive alternative exons, evidence for widespread antagonistic regulation was not observed (Du et al. 2010).

CELF proteins are present in both cytoplasm and nucleus and play roles in deadenylation, RNA stability, and translation, as well as splicing (Paillard et al. 2003; Timchenko et al. 2004; Vlasova et al. 2008). Tethering CELF1 to an mRNA is sufficient for destabilization of the mRNA (Barreau et al. 2006), and addition of GU-rich sequences to an RNA confers destabilization following *CELF1* overexpression (Masuda et al. 2012). Human CELF1 is 88% identical to the *Xenopus* homolog embryo deadenylation element binding protein (EDEN-BP) and can functionally replace it, binding to the EDEN element to control poly(A) tail length and aiding in rapid deadenylation of maternal mRNAs after fertilization (Paillard et al. 2003). CELF1 interacts with poly(A)-specific ribonuclease (PARN) in HeLa cell extracts to promote deadenylation of *FOS* and *TNF* transcripts (Moraes et al. 2006). Furthermore, siRNA-mediated knockdown of *CELF1* in HeLa cells and myoblasts led to the stabilization of a set of normally rapidly degraded transcripts bound by CELF at GU-rich elements (GREs) and GU-repeats (Vlasova et al. 2008; Ji et al. 2009; Lee et al. 2010; Rattenbacher et al. 2010). It is possible that abnormal up-regulation of CELF proteins in adult tissues in DM1 could impact the stability of many transcripts and contribute to DM pathology.

To investigate the functional significance of the post-natal down-regulation of CELF in heart and skeletal muscle tissues, we inducibly expressed *CELF1* in adult mouse heart or muscle or *CELF2* in adult heart and performed a time-series RNA-seq analysis following induction, complemented by high-throughput biochemical assays and comparisons to available data for MBNL1. Our observations lead to a model in which CELF and MBNL proteins compete to specify different mRNA fates so as to change the expression and localization of hundreds of mRNAs during development.

## Results

### Identification of hundreds of *CELF*-responsive exons

We performed strand-specific paired-end RNA sequencing (RNA-seq) of poly(A)^+^ RNA from skeletal muscle or heart of mice in which *CELF1* was induced, or heart of mice in which *CELF2* was induced at several time points post-induction, and from control mice, in biological triplicate. The *CELF1* mice have been described previously (Koshelev et al. 2010; Ward et al. 2010). The *CELF2* mouse model was newly developed here and is described in Methods. All mice used are summarized in Supplemental Table S1. Human *CELF1* or *CELF2* transgenes were induced in mice by administration of doxycycline, in separate mouse lines for each tissue/protein pair, and reached levels between five- and 10-fold above endogenous levels (Supplemental Fig. S1A,B). The level of *CELF1* mRNA increased strongly before declining at the end of the time course, likely as a secondary effect of heart pathology on the *Myh1* promoter that drives *rtTA* (Lowes et al. 1997). However, CELF1 protein levels consistently increased in these mice at least eightfold in skeletal muscle (Ward et al. 2010) and approximately fourfold in heart and remained high through at least day 7 (Koshelev et al. 2010). Heart pathology was not observed in the *CELF2* mouse, and mRNA and protein levels increased throughout the time course (Supplemental Fig. S1C,F). RNA was isolated from three Tet-inducible mice each at 12, 24, and 72 h and 7 d following induction and from three control mice lacking Tet-inducible *CELF1* or *CELF2* transgenes, treated with doxycycline for 72 h. The MISO software for statistical inference of splicing changes using RNA-seq data (Katz et al. 2010) was used to estimate a percentage spliced in (PSI, or Ψ) value for each cassette (skipped) exon, which estimates the fraction of a gene's messages that include the exon. We also calculated Ψ values for alternative 5′ and 3′ splice sites and retained introns. For each comparison of Ψ between samples, we calculated a Bayes factor (BF) representing the ratio of the likelihood of the hypothesis that Ψ values differ from the null hypothesis of no change in Ψ, and we used BFs to identify differentially regulated exons.

**Figure 1.**
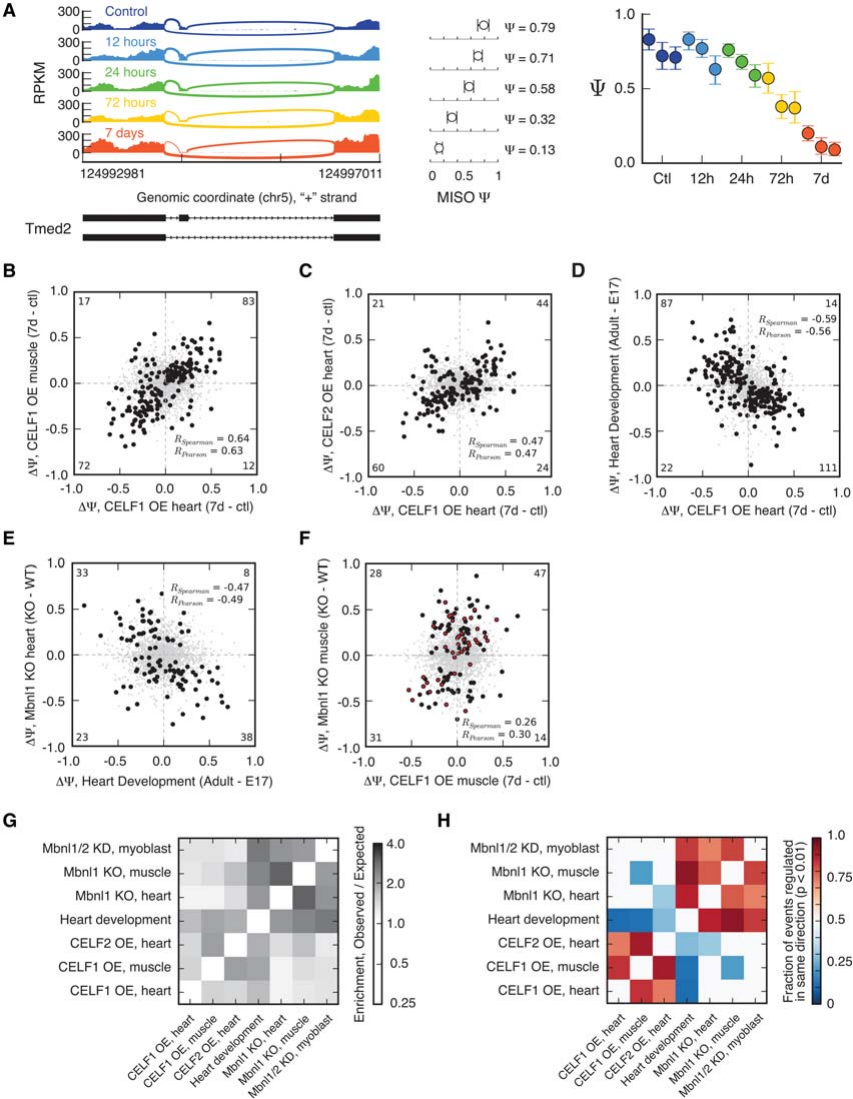
*CELF1* and *CELF2* regulate hundreds of splicing events, reversing many changes during heart development and antagonizing a subset of *Mbnl*-regulated exons. (*A*) RNA-seq read coverage across *Tmed2* exon 3 from mouse heart at several time points following *CELF1* induction. MISO Ψ values and 95% confidence intervals shown at *right*. (*B*) Splicing changes that occur following *CELF1* overexpression (OE) in heart correlate with splicing changes that occur following *CELF1* overexpression in muscle. *n* = 2496 alternative splicing events (skipped exons, alternative 3′ splice sites, alternative 5′ splice sites, retained introns, and mutually exclusive exons) shown: Monotonically changing exons shown as black circles, others as gray dots. Correlation values of monotonic events (shown) are higher than for all events: The numbers of monotonic events in each quadrant are shown in corners. (*C*) Splicing changes that occur in response to *CELF1* overexpression in heart correlate with splicing changes that occur in response to *CELF2* overexpression in heart. As in *B*, with *n* = 2129 skipped exons shown. (*D*) Splicing changes that occur in response to *CELF1* overexpression in heart inversely correlate with splicing changes that occur during mouse heart development. As in *B*, with *n* = 1952 skipped exons shown. (*E*) Splicing changes that occur in *Mbnl1* KO heart inversely correlate with splicing changes that occur during mouse heart development. *n* = 3190 skipped exons shown, as in *B*. (*F*) Splicing changes that occur in *Mbnl1* KO muscle correlate with splicing changes that occur following *CELF1* overexpression in muscle. *n* = 1501 skipped exons shown. Events that changed monotonically, or with BF > 5, following *CELF1* overexpression in muscle or *Mbnl1* KO in muscle, respectively, are shown in black, and correlations are listed for these events. Events that also changed monotonically during heart development are shown in red. (*G*) Exons regulated during heart development also tend to change in *Mbnl1* and *Mbnl2* knockdown myoblasts, in *Mbnl1* and *Mbnl2* knockout mice, and in *CELF1* or *CELF2* overexpressing mice. Enrichment (observed/expected number of regulated exons) is shown in the heatmap. (*H*) Splicing of exons regulated in response to *CELF* overexpression or *Mbnl* depletion tends to change in a direction opposite from changes that occur during heart development. The fraction of events changing in the same direction for each pair of comparisons is shown in the heatmap (only biases significant at *P* < 0.01 by binomial test are colored). See also Supplemental Figures S1 and S2 and Supplemental Tables S1–S4.

We identified thousands of exons whose Ψ values changed between time points at a BF cutoff of five, a value supported by previous studies of RBPs (Katz et al. 2010). For example, exon 3 in *Tmed2* had a Ψ of 79% in the control heart but decreased to 13% after *CELF1* induction for 7 d (Fig. 1A). To identify splicing events that change monotonically over time, we developed a permutation-based method that could be applied to time course data. We ordered samples chronologically, and for each event compared all pairs of samples from different time points, tallying the number of comparisons representing a significant increase or significant decrease in Ψ (at BF > 5). We calculated a quantity called δ, the number of significant positive ΔΨ values (increases over time) minus the number of significant negative ΔΨ values (Supplemental Fig. S2A). To assess statistical significance, we recalculated δ after randomly permuting the sample labels. Repeating this process 100 times, we generated a null distribution and derived a “monotonicity *Z*-score” (MZ) defined as MZ = (δ − μ)/σ, where μ and σ are the mean and standard deviation of the null distribution of δ values, respectively. Large positive or negative MZ values indicate consistent increase or decrease, respectively, in the inclusion of an alternative exon over the time course. (MZ scores and Ψ values of alternative exons for all experiments are listed in Supplemental Table S2.) We observed 627 and 825 exons responsive to *CELF1* induction at a MZ score of 1.8 in heart and skeletal muscle, respectively.

### About 30% of splicing changes in heart development respond to *CELF1* induction

We next sought to understand the functions of CELF proteins in different tissues and developmental stages. By comparing ΔΨ values of monotonically changing (high MZ) skipped exons between *CELF1*-induced heart and *CELF1*-induced skeletal muscle, we observed a high correlation (*R*_Spearman_ = 0.64), suggesting that the functions and regulatory targets of CELF1 in these tissues are quite similar (Fig. 1B). To ask about functions of different CELF family members, we compared splicing changes in the heart following induction of *CELF1* or *CELF2*. This comparison yielded a moderate positive correlation (*R*_Sp_ = 0.47), suggesting similar but not necessarily identical splicing functions (Fig. 1C).

To explore connections to the developmental roles of CELF proteins, we compared splicing in the *CELF1* heart to changes that occur during normal heart development. Developmental changes were analyzed by use of an RNA-seq time series (Giudice et al. 2014), including embryonic day 17 (E17), post-natal (PN) day 1, PN 10, PN 28, and adult, during which CELF1 protein levels fall by more than 10-fold (Kalsotra et al. 2008). In all, 234 alternatively skipped exons changed monotonically in heart development and/or following *CELF1* induction. Splicing of 198 (85%) of these exons changed in the opposite direction following *CELF1* induction from that during heart development, with a fairly strong negative correlation (*R*_Sp_ = −0.59, Fig. 1D). Thus, *CELF1* induction triggers widespread reversal of heart developmental splicing and suggests that normal reductions in CELF activity during heart development may contribute to a substantial portion—perhaps 30% (Supplemental Fig. S2B)—of the splicing changes that occur during normal heart development (Kalsotra et al. 2008; Giudice et al. 2014).

### More than 200 exons are regulated antagonistically by CELF and MBNL proteins

Six mouse exons (Kalsotra et al. 2008) and at least one human exon (Ho et al. 2004) are known to be regulated antagonistically by CELF1 and MBNL1. Confirming and extending this trend, we identified dozens of additional exons regulated by both factors. *Mbnl1* depletion (Wang et al. 2012) mimicked a reversal of many splicing changes that occur during heart development (*R*_Sp_ = −0.47) (Fig. 1E). In skeletal muscle, we detected 120 exons whose splicing was responsive to both proteins, of which 78 (65%) were regulated in an antagonistic fashion (Fig. 1F; for exons responsive to both factors, see Supplemental Table S3). Extending this analysis to additional tissues and cell lines and comparing to heart development, we found that exons responsive to induction of *CELF1* or *CELF2* showed a strong and quite general tendency to also respond to *Mbnl1* depletion and to be developmentally regulated (Fig. 1G). Furthermore, the direction of splicing regulation tended to oppose normal developmental transitions, with both *Mbnl1* depletion and *CELF1* induction often reversing developmental changes (Fig. 1H; Supplemental Table S3).

The observed splicing antagonism might result from direct effects of CELF1 on splicing or potentially from indirect effects on MBNL expression by CELF proteins. We found that *CELF1* induction in heart and muscle yielded only modest changes in the mRNA levels of *Mbnl1* (up 15% in heart, down 10% in muscle) and *Mbnl2* (up 9% in heart, down 12% in muscle) by RNA-seq RPKM and did not change inclusion of *Mbnl1* exon 5, which can regulate nuclear-cytoplasmic distribution of MBNL1 (Supplemental Fig. S1D,E; Lin et al. 2006). Furthermore, *CELF1* overexpression in C2C12 mouse myoblasts did not detectably alter MBNL1 protein levels (Supplemental Fig. S1G) or change the localization of MBNL1 by Western blot (data not shown). Finally, inclusion levels of exons bound and regulated by MBNL1, but not bound by CELF1, showed no tendency to change in a direction consistent with an overall decrease in MBNL1 activity (data not shown). These observations indicate that antagonistic regulation of many MBNL-dependent splicing events by CELF proteins results predominantly from direct effects on splicing rather than indirectly through regulation of MBNL activity.

In total, 206 alternative exons were responsive to both *Mbnl1* depletion and *CELF1* induction in muscle and/or heart. These exons were enriched for several Gene Ontology (GO) categories, including “cell differentiation,” “multicellular organismal development,” “microtubule cytoskeleton,” and “cell junction,” suggesting roles in developmental remodeling of the heart (Supplemental Table S4). Furthermore, 124 of these exons (60%) preserved the translational reading frame (compared to a background of 44% of alternative exons, *P* < 0.001, binomial test), suggesting that the splicing activities of CELF and MBNL proteins are predominantly involved in shaping the spectrum of protein isoforms expressed in the heart. These observations provide genome-wide evidence for the principle that CELF and MBNL proteins exert opposing effects on the splicing of a large cohort of exons. They also expand the number of developmentally regulated CELF-/MBNL-responsive exons several-fold.

### Transcriptome-wide binding locations of CELF1 in heart, muscle, and myoblasts

To identify transcript sites bound by CELF1, we performed CLIP-seq analysis using the 3B1 mouse monoclonal antibody against the endogenous CELF1 protein, yielding 1.6 million, 1.0 million, and 1.6 million reads uniquely mapping to the genome and splice junctions in 16-wk-old C57BL/6 heart, 16-wk-old C57BL/6 muscle, and C2C12 myoblast samples, respectively, after collapsing identical reads. Mapping occurred predominantly to transcribed regions, with enrichment for introns and/or 3′ UTRs (Supplemental Fig. S3A), consistent with previous studies (Masuda et al. 2012).

**Figure 2.**
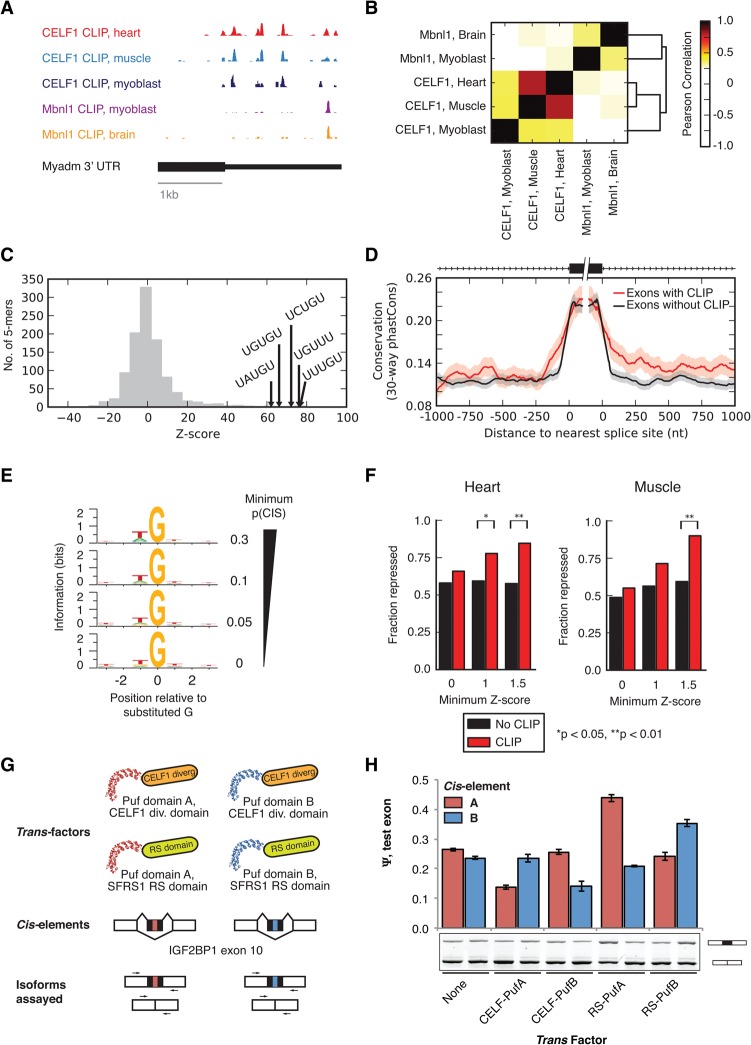
CELF1 binds to consistent locations across cells and tissues and represses splicing of bound exons. (*A*) CELF1 CLIP-seq read coverage across the 3′ UTR of the mouse *Myadm* gene. Binding locations are highly correlated between muscle and heart. (*B*) Correlation of CLIP tag densities in 5-nt windows across all 3′ UTRs expressed in mouse heart, muscle, and myoblasts. (*C*) Histogram of enrichment *Z*-scores of 5mers based on frequency of occurrence in CELF1 heart CLIP clusters relative to control regions from the 3′ UTRs. (*D*) Meta-exon analysis of conservation (mean + 95% confidence interval of phastCons score in 5-nt windows shown) at a range of distances from 3′ and 5′ splice sites of exons with (red) and without (black) overlapping CELF1 CLIP clusters in heart. *n* = 432 skipped exons shown. (*E*) Information content (relative entropy compared to uniform) of genomic positions in regions where CELF1 CLIP-seq reads map, grouped by the frequency of substitution in CLIP reads relative to genome of the central G position. (*F*) Fraction of significantly repressed exons (Ψ at 7 d < Ψ in control animals) with or without CLIP clusters, at three thresholds of monotonicity *Z*-score from *CELF1* overexpression time courses in heart and muscle. Significance was assessed by binomial test, where the number of events with CLIP clusters that were repressed was compared to the fraction of events without CLIP clusters that were repressed. (*G*) Design of experiment involving splicing reporters and Pumilio-based synthetic splicing factors, as well as assessment of splicing by qRT-PCR. (*H*) Tethering the divergent domain of CELF1 to a cassette exon in a splicing reporter by Pumilio fusion promotes exon skipping. In a positive control, tethering of RS domains to the cassette exon enhances exon inclusion. Enhancement by RS and repression by CELF1 occur only when the Pumilio domain has affinity for the inserted oligonucleotide. See also Supplemental Figures S3 and S4 and Supplemental Table S5.

CELF1 binding locations were consistently observed across different tissues, for example, in the 3′ UTR of the myeloid-associated differentiation marker gene, *Myadm* (Fig. 2A). CELF1 binding density along mRNAs, assessed in 5-nucleotide (nt) windows, was highly correlated across tissues and cell lines and was distinct from that observed for MBNL1 (Fig. 2B). Controlling for pre-mRNA length and gene expression, CELF1 CLIP clusters were enriched for UGU-containing pentanucleotides (5mers) in the heart and other tissues (Fig. 2C; Supplemental Fig. S3B). Introns flanking alternative exons with CLIP clusters within 1 kb from either splice site were more highly conserved across species, in particular in the downstream intron, supporting their in vivo function (Fig. 2D).

To more precisely map sites of CELF1 binding, we measured the frequency of “crosslink-induced substitutions” (CISs)—positions where CLIP-seq reads differ from the genome (Kishore et al. 2011; Wang et al. 2012)—at each position within CLIP clusters. We noted that guanines with a high CIS showed biases in flanking bases, with the −1 base preceding the substituted guanine increasingly biased toward uracil as CIS frequency increased (reaching ∼61% in heart); the base at the +1 position was also biased toward uracil (Fig. 2E; Supplemental Fig. S3C). The implied CIS-enriched motif UGU resembles the motifs observed in Figure 2C and in previous studies of CELF1 binding affinity (Marquis et al. 2006; Lambert et al. 2014), suggesting that frequently substituted guanines are highly enriched for sites of direct crosslinking to CELF1 protein.

### Context-dependent regulation of splicing by CELF1

Exons whose splicing changed after *CELF1* induction were enriched for CELF1 CLIP clusters (Supplemental Fig. S4). To determine an RNA map describing the splicing regulatory activity of CELF1, we analyzed the location of CELF1 clusters relative to exons responsive to *CELF1* overexpression in heart and/or muscle. We found that cassette alternative exons bound by CELF1 were more likely to be repressed following *CELF1* induction in heart and muscle (Fig. 2F), with 80%–85% of exons with MZ score >1.5 being repressed versus 15%–20% being activated. Though we had reasonable statistical power, we did not observe a consistent trend toward repression or activation of exons bound by CELF1 in the upstream or downstream introns. These observations suggest that exonic binding by CELF family members may directly repress splicing, while the direction of splicing regulation resulting from intronic binding may depend on other variables such as RNA structure or cooperation or antagonism with other RBPs (Dembowski and Grabowski 2009; Goo and Cooper 2009).

To directly test whether CELF1 could repress splicing when bound to a cassette exon, we fused the C-terminal “divergent domain” of CELF1 (omitting the RRMs) to two different Pumilio (Puf) domains, each recognizing a distinct eight-base Puf motif, as in Wang et al. (2009a). We expressed these constructs in HeLa cells, along with a splicing reporter (Xiao et al. 2009) containing either cognate or noncognate Puf motifs within the cassette exon, and used quantitative RT-PCR to assess splicing changes (Fig. 2G). We found that expression of CELF1-Puf fusion decreased Ψ from ∼25% to ∼12% when paired with the cognate Puf motif but had a negligible effect when paired with the noncognate motif. Thus, recruitment of a single CELF1 protein molecule to an exon appears sufficient to reduce splicing by double-digit percentage values in this system. Natural regulatory targets often have multiple CELF1 binding sites; recruitment of multiple CELF1 proteins is likely to magnify the impact on splicing in the mode of other splicing regulatory factors (Matlin et al. 2005). Control RS domain-Puf fusions activated splicing, as expected (Fig. 2H; Wang et al. 2009a). These observations indicate that tethering the CELF1 divergent domain to an exon is sufficient to repress its splicing.

### Dose-dependent down-regulation of expression associated with CELF1 binding to 3′ UTRs

**Figure 3.**
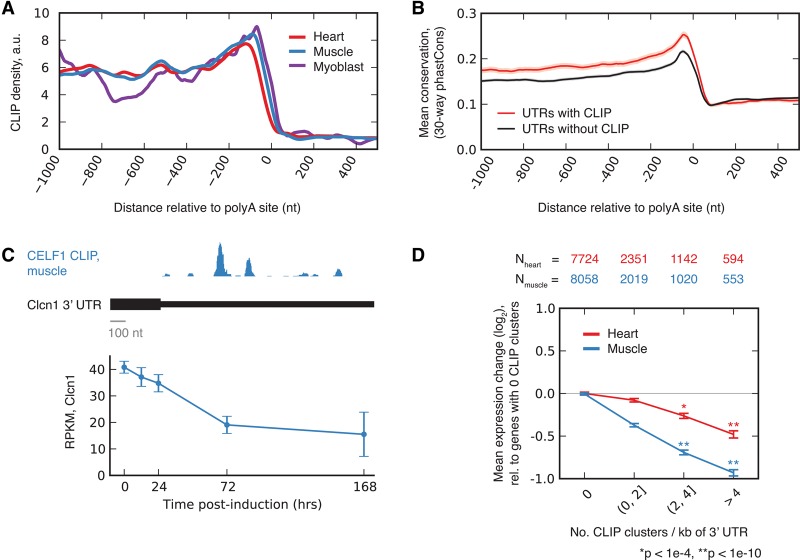
CELF1 binds to 3′ UTRs and regulates message stability in a dose-dependent fashion. (*A*) Mean CELF1 CLIP density at positions along 3′ UTRs in heart, muscle, and myoblasts. (*B*) Mean conservation in sets of 3′ UTRs with and without CELF1 CLIP clusters with similar expression levels and UTR lengths (shading represents SEM). (*C*) Expression of *Clcn1* in muscle based on RNA-seq (mean ± SD) at various times following *CELF1* overexpression (*bottom*); CLIP density in *Clcn1* 3′ UTR (*top*). (*D*) Mean log expression change following *CELF1* induction (7 d over control) for transcripts grouped by number of CELF1 CLIP clusters in their 3′ UTRs. Transcripts with greater CLIP cluster density are down-regulated more strongly in heart and muscle (number of genes in each category listed *above*). Significance was assessed by rank-sum test, where each CLIP cluster bin was compared to the zero CLIP cluster bin. See also Supplemental Table S6.

We observed extensive CELF1 binding to 3′ UTRs, with increased density upstream of the cleavage and polyadenylation site (PAS) (Fig. 3A). Consistent with the established activity of CELF1 in binding mRNAs and recruiting cytoplasmic deadenylases (Vlasova-St Louis et al. 2013), binding density increased close to the PAS on the upstream side and fell to background levels just downstream from the PAS site in heart, muscle, and myoblast. Increased phylogenetic conservation was observed for 3′ UTRs containing CELF1 CLIP clusters, relative to 3′ UTRs with a similar length and expression level, suggesting that these mRNAs are enriched for conserved functional motifs or structures (Fig. 3B). GO analysis of 3′ UTR binding targets of CELF1 revealed enrichment of proteins related to muscle structures, such as M band and I band, and factors involved in vesicle and protein transport in both heart and muscle (Supplemental Table S5).

Analyzing gene expression globally, we observed that mean expression of CELF1-bound mRNAs decreased substantially at 7 d after *CELF1* induction relative to genes not bound by CELF1 (Supplemental Table S6). For example, the *Clcn1* mRNA, which had prominent CELF1 binding clusters in its 3′ UTR, decreased in expression 50%–70% from its initial level 7 d after *CELF1* induction, in both muscle (Fig. 3C) and heart (Supplemental Table S6). Furthermore, the average magnitude of target down-regulation increased monotonically with the density of CLIP clusters in the 3′ UTR, reaching 1.4-fold and twofold in heart and muscle, respectively, for messages with four or more CLIP clusters per kb of 3′ UTR (Fig. 3D). The consistent association between binding and down-regulation and the “dose-response” effect observed in Figure 3D both support a direct role for CELF1 in target mRNA repression.

We expected similar levels of *CELF1*-mediated target repression to occur in heart and muscle, given that the level of *CELF1* induction was similar in both tissues (Supplemental Fig. S1; Koshelev et al. 2010; Ward et al. 2010). However, the magnitude of target down-regulation in the heart was consistently only about half of that observed in muscle for messages with similar CLIP density. This difference might be related to a pattern we observed in which targets of miRNAs were derepressed in the heart following CELF1 induction (Supplemental Fig. S5).

### CELF1 binding to alternative 3′ UTRs is associated with isoform-specific repression

**Figure 4.**
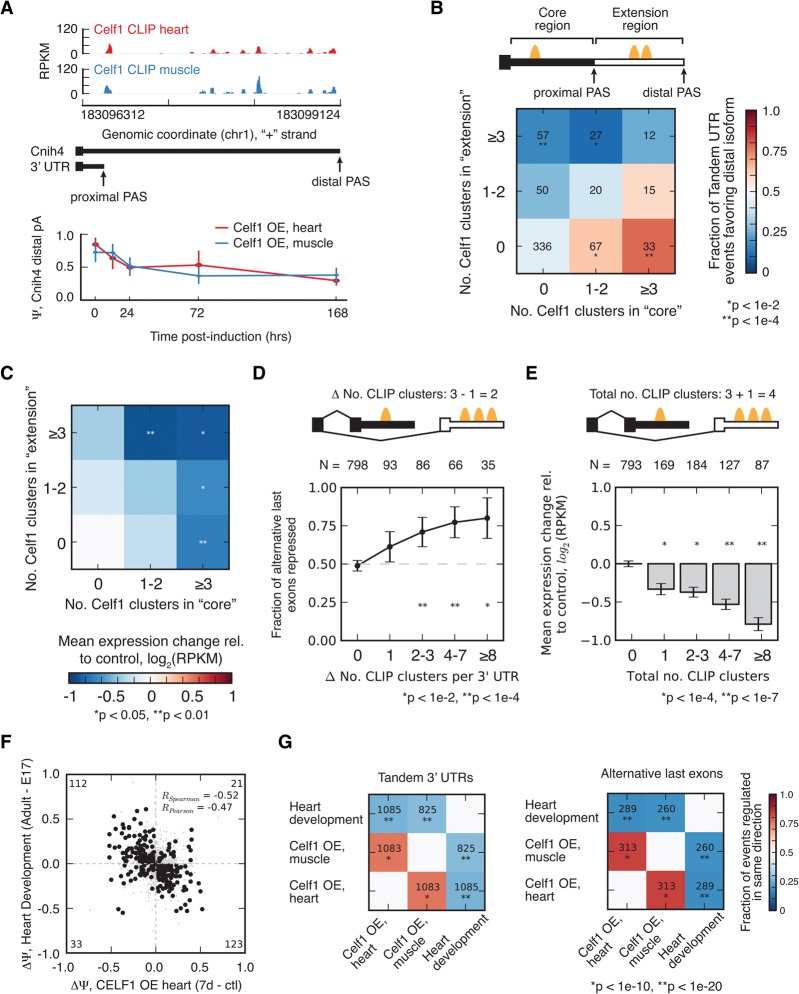
*CELF1* regulates abundance of bound alternative 3′ UTRs, reversing developmental changes. (*A*) CELF1 CLIP-seq density in 3′ UTR of the *Cnih4* gene, which has two alternative PASs whose relative abundance changes following *CELF1* overexpression and during heart development. (*B*) *CELF1* regulates tandem 3′ UTR events in a manner that is dependent on the number of binding sites in the core and/or extension region of the 3′ UTR. The heatmap shows the fraction of tandem UTR events biased toward usage of the proximal PAS following *CELF1* overexpression in muscle for events grouped by the number of CLIP clusters in the proximal or distal region of the 3′ UTR (MZ > 1.6). Significance was assessed by binomial test, assuming equal likelihood for usage of long or short isoforms. (*C*) Messages with regulated tandem 3′ UTR events tend to be down-regulated following *CELF1* induction. The heatmap shows the mean expression change following *CELF1* induction in muscle for genes harboring events grouped by the number of CLIP clusters in the proximal or distal region of the 3′ UTR. Significance was assessed by rank-sum test, where each bin was compared to the bin with zero CLIP clusters. (*D*) *CELF1* regulates ALE expression in a manner that is dependent on the number of binding sites in the competing 3′ UTRs. The fraction of ALEs that is significantly repressed following *CELF1* overexpression in muscle, grouped by density in repressed 3′ UTR minus density in enhanced 3′ UTR (MZ > 1.5). Significance was assessed by binomial test, assuming equal likelihood for usage of each ALE isoform. (*E*) Messages with CELF1 binding within ALEs tend to be down-regulated following *CELF1* induction. (Bar plot) Mean expression change following *CELF1* induction in muscle for genes with varying numbers of CLIP clusters within ALEs. Significance was assessed by rank-sum test relative to genes with no CLIP clusters. (*F*) Changes in ALE usage during heart development inversely correlate with those that occur in response to *CELF1* overexpression in heart. Correlation coefficients shown for events meeting a minimum *Z*-score threshold (heart development, 1.4; *CELF1* OE, 1.8), as in Figure 1B. (*G*) Spearman correlation coefficients and significance of correlation are displayed in heatmap format for change in ALE usage (*right*) or change in tandem 3′ UTR usage (*left*) for pairwise comparisons of isoform changes during heart development, *CELF1* overexpression in heart, and *CELF1* overexpression in muscle. See also Supplemental Figure S6.

Many mammalian genes end with more than one PAS, yielding “tandem UTR” isoforms, differing in the length of their 3′ UTRs. We observed many instances of altered 3′ UTR isoform abundance following *CELF1* induction in the heart and muscle. For example, the *Cnih4* gene, a member of the cornichon family AMPA receptor auxiliary proteins involved in the selective transport and maturation of TGF-alpha family proteins, contains multiple tandem alternative PASs that are used in these tissues. CELF1 CLIP clusters are located in the extension region of the *Cnih4* 3′ UTR between the two PASs (Fig. 4A). The relative abundance of the longer isoform relative to total *Cnih4* mRNAs was ∼85% in adult heart and ∼75% in muscle but decreased monotonically in both tissues following *CELF1* induction to ∼30% and 40%, respectively (Fig. 4A), consistent with CELF1-mediated down-regulation of the longer isoform. Our genome-wide analysis revealed that 57 tandem UTR events contained at least three CELF1 clusters in the extension region and zero clusters within the core (shared) region; of these, ∼75% followed the pattern of *Cnih4*, exhibiting decreased abundance of the longer isoform relative to the shorter isoform following *CELF1* induction. Across other combinations of binding sites, a consistent trend was observed with higher relative abundance of CELF1 clusters in extension versus core regions associated with greater relative decline in distal isoform expression (Fig. 4B).

Changes in tandem UTR expression associated with CELF1 binding could, in principle, result from regulation of cleavage and polyadenylation to produce less of the distal PAS isoform or by differential regulation of the stabilities of the isoforms. If regulation occurs by the latter mechanism, overall gene expression should decrease following *CELF1* induction in proportion to the extent of binding in the core and extension regions, with binding in the core exerting a stronger effect, since the core region is shared by both isoforms. Our analysis of expression changes of tandem UTR genes revealed exactly this: Following induction, gene expression decreased with the number of CELF1 CLIP clusters in either region (Fig. 4C). By using a linear model, we compared the magnitude of down-regulation associated with extension and core sites and found that sites in the core were associated with down-regulation of mRNA expression by 6.9 ± 2.7% (95% CI) per site, and sites in the extension were associated with 2.7 ± 2.5% down-regulation. Because of their presence in both long and short isoforms, core sites are expected to exert stronger effects on overall gene expression than are sites in the extension, as observed. Therefore, the effects of CELF1 on tandem UTR isoform expression can be explained based on the factor's known ability to destabilize mRNAs via recruitment of deadenylases without needing to invoke a potential activity in regulating cleavage and polyadenylation.

In thousands of other mammalian genes, a combination of alternative splicing and alternative PAS usage gives rise to alternative last exons (ALEs) (Fig. 4D). The distinct 3′ UTRs of ALEs provide natural reporters of 3′ UTR functions, since they generally have little or no effect on the ORF. For more than 1000 genes, we observed a monotonic change in the relative expression of ALE isoforms following *CELF1* induction in muscle. The ALE isoform exhibiting greater CELF1 binding tended to be down-regulated, and the magnitude of this bias increased as the difference in the number of sites increased (Fig. 4D). A similar bias was observed in ALE pairs lacking CELF1 binding within 500 nt of each alternative splice site (Supplemental Fig. S6), suggesting that changes in ALE abundance do not commonly result from regulation of splicing by CELF1. Furthermore, as was observed for tandem UTRs, increased CELF1 binding was associated with increased mRNA down-regulation (Fig. 4E), with an average magnitude of 5.9 ± 1% (95% CI) per CLIP cluster, consistent with mRNA destabilizing activity. Together, the consistent pattern of down-regulation of mRNAs (Fig. 3D) and of specific mRNA isoforms (Fig. 4B–E) associated with CELF1 binding to 3′ UTRs provides evidence of widespread direct effects of binding on mRNA expression and identifies numerous instances of gene- and isoform-specific regulation.

### Evidence for CELF1-mediated regulation of alternative 3′ UTR isoforms in development

The dramatic changes in CELF1 levels that occur during heart development led us to ask whether these 3′ UTR isoforms are often developmentally regulated. By comparing changes in 3′ UTR isoform abundance during heart development to changes following *CELF1* induction in heart, we observed a strong negative correlation (*R*_Sp_ = −0.52) (Fig. 4F). Therefore, as observed at the level of splicing, *CELF1* induction tends to reverse isoform changes that occur during normal heart development. This negative correlation held whether analyzing *CELF1* induction in heart or skeletal muscle and whether analyzing tandem UTRs or ALEs (Fig. 4G). The hundreds of 3′ UTR isoforms involved in these patterns suggest that developmental reductions in CELF1 activity underlie a substantial fraction of 3′ UTR isoform changes that occur during heart development.

### Antagonistic regulation of mRNA expression by CELF and MBNL proteins

**Figure 5.**
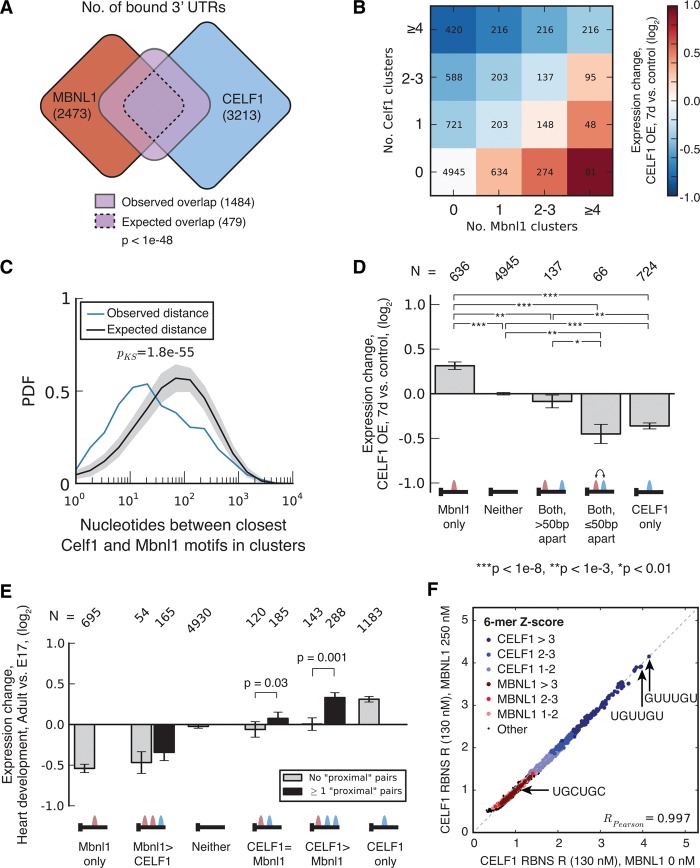
CELF1 and MBNL1 bind in close proximity to the same 3′ UTRs and exert opposing effects on mRNA stability. (*A*) Venn diagram showing the expected and observed overlap between CELF1 and MBNL1 3′ UTR targets (CELF1 data from muscle, MBNL1 data from myoblasts). The observed overlap is approximately three times larger than expected (analysis controlled for gene expression) and significant by Fisher's exact test. (*B*) Expression change following *CELF1* induction in muscle (7 d versus control) for transcripts grouped by number of MBNL1 and CELF1 CLIP clusters in the 3′ UTR. CLIP clusters for MBNL1 and CELF1 were derived from myoblast and muscle, respectively. (*C*) The probability density function (PDF) of the distribution of distances between CELF1 CLIP clusters in muscle and MBNL1 CLIP clusters in myoblasts, in 3′ UTRs with binding for both proteins. Distances for true binding sites are shown in blue; for randomly placed binding sites, in black. Statistical significance was assessed by modified KS test, and the distribution of distances in shuffled controls is shown in gray. (*D*) Expression change following CELF1 induction in muscle (7 d versus control) for transcripts with exactly one MBNL1 CLIP cluster and exactly one CELF1 CLIP cluster. Genes were grouped according to whether the distance between motifs is less than or greater than 50 base pairs (bp). Significance was assessed by rank-sum test. (*E*) Expression change during heart development (adult versus E17) for transcripts with varying numbers of MBNL1 and CELF1 CLIP clusters. Genes were grouped according to the relative abundance of MBNL1 and CELF1 CLIP clusters and by the presence/absence of a proximal MBNL1/CELF1 binding pair. Only transcripts with gene expression MZ scores >0.5 were included in this analysis, and significance was assessed by KS test. See also Supplemental Figures S7 and S8 and Supplemental Tables S7 and S8. (*F*) MBNL1 presence does not affect in vitro binding of CELF1 to RNA. 130-nM tagged CELF1 and 250-nM MBNL1 were equilibrated in vitro with random RNA 40mers in an RNA Bind-n-Seq experiment. The tagged CELF1 was pulled down, bound 40mers were eluted and sequenced, and RBNS *R* of each 6mer was calculated as the frequency in the pulldown library divided by the frequency in the input library. 6mers were classified as weak, medium, or strong (RBNS *R Z*-score between 1–2, 2–3, or >3, respectively), CELF1-binding, MBNL-binding, or none of the above (“Other”) using the data from Lambert et al. (2014) and color-coded as indicated.

In addition to their nuclear functions in RNA splicing, MBNL proteins also exert cytoplasmic functions, targeting 3′ UTR-bound mRNAs for localization to membrane destinations and promoting translation of targeted messages (Adereth et al. 2005; Wang et al. 2012). To ask whether this function might be related to mRNA repression by CELF proteins, we compared CELF1 and MBNL1 3′ UTR binding targets. We observed more than 1000 3′ UTRs bound by both CELF1 and MBNL1 (with at least two CLIP clusters for each factor), three times as many as expected by chance (*P* < 1 × 10^−48^, Fisher's exact test) (Fig. 5A; for CLIP clusters, see Supplemental Table S7). Given that gene expression level impacts the statistical power to detect CLIP binding, we assessed the fold enrichment of cobinding relative to independence in sets of genes binned by expression level, and observed as well as expected numbers of shared targets are shown in Figure 5A. These genes were enriched for a number of GO categories, including categories related to cytoskeleton, such as “actin cytoskeleton organization,” and to development, such as “mesoderm formation,” “patterning of blood vessels,” “ventricular septum development,” and other categories (Supplemental Table S8), suggesting important developmental functions. These observations led us to explore whether CELF and MBNL might exert antagonistic, or synergistic, effects on mRNA levels.

To assess possible functional relationships between the effects of CELF and MBNL proteins on expression, we assessed gene expression changes for sets of genes grouped by extent of CELF1 and MBNL1 binding. Expression changes following *CELF1* induction exhibited a strong dependence on the ratio of CELF1 to MBNL1 binding sites: Greater CELF1 binding was associated with strong (up to approximately twofold) average down-regulation, messages with similar numbers of CELF1 and MBNL1 clusters exhibited little change, and those with greater MBNL1 binding showed increased expression (Fig. 5B). These data suggest that MBNL binding can protect a message from CELF-mediated repression. The highly consistent pattern in which each additional CELF binding site conferred reduced expression, while each additional MBNL binding site conferred increased expression, suggests that MBNL1 and CELF1 compete for cobound mRNAs to target them for different fates. The set of mRNAs that (1) were down-regulated upon *CELF1* induction; (2) are bound by both CELF1 and MBNL1; and (3) changed their localization upon *Mbnl* depletion in the expected direction (away from membrane and toward the insoluble compartment, based on our previous study) (Wang et al. 2012) included *Cpe*, *Igfbp5*, *Kcnj2*, and *Sobp* (see Discussion).

Within the set of mRNAs not detectably bound by CELF1, the up-regulation of mRNAs bound more strongly by MBNL1 relative to less-bound messages was unexpected. One possible explanation is that this signal derives from CELF1 CLIP false negatives, i.e., messages that are bound by CELF1 but that failed to crosslink efficiently (or failed to be recovered in our CLIP-seq data for other technical reasons). Another possibility is that CELF proteins may exert a general nonspecific or low-specificity, mRNA decay-promoting activity at high levels and that MBNL binding protects against this effect as well as the effects of specific CELF1 binding.

To further explore the relationship between CELF1 and MBNL1 proteins, we analyzed the relative locations of CELF1 and MBNL1 binding sites in 3′ UTRs. We found that sites bound by CELF1 and MBNL1 tended to cluster together more than expected, relative to randomly placed clusters (*P* < 1.8 × 10^−55^ by Kolmogorov-Smirnov [KS] test) (Fig. 5C). This proximity effect remained highly significant when performing additional analyses that preserved the locations of CELF1 and MBNL1 binding sites but permuted protein identity, a treatment that controls for variations in local base composition and accessibility along mRNAs (Supplemental Fig. S7A).

To ask whether functional antagonism between MBNL1 and CELF1 might depend on binding site proximity, we examined the effects of *CELF1* induction on messages with proximal (≤50 nt) or distal (>50 nt) pairs of CELF1 and MBNL1 binding sites. The presence of a distal MBNL1 site was sufficient to abrogate the repression normally associated with presence of a CELF1 site, but presence of a proximal MBNL1 site lacked such a protective effect (Fig. 5D). These trends extended to messages containing multiple CELF1 sites. Comparing sets of mRNAs with the same distributions of CELF1 site counts and with exactly one MBNL1 site, those with a greater number of proximal CELF1/MBNL1 pairs were more strongly down-regulated following *CELF1* induction (Supplemental Fig. S5B). This phenomenon of “local inhibition” of MBNL1's activity by CELF1 suggests that CELF1 can bind to messages when MBNL1 is bound nearby, and is readily explicable by either of two models: (1) “local inhibition of binding,” in which inhibition results simply from CELF1 inhibiting nearby binding of MBNL1, either directly through steric hindrance or indirectly via effects on local RNA structure; or (2) “local inhibition of function,” in which occupancy of CELF1 binding sites proximal to bound MBNL1 antagonizes the localization/stabilization function conferred by MBNL1 without affecting MBNL1's binding.

Under either model, mRNAs containing a proximal pair of MBNL and CELF sites are expected to be more responsive to developmental changes in CELF and MBNL levels than messages containing distal pairs of sites. To explore this idea, we assessed developmental changes in expression of mRNAs grouped by binding site count and presence of proximal or distal CELF/MBNL site pairs (Fig. 5E). Overall, we observed moderate derepression in proportion to the number of CELF sites present and observed repression when MBNL sites exceeded CELF sites. This pattern reverses the pattern observed in Figure 4B, as expected given the down-regulation of CELF1 during heart development. Moreover, messages containing proximal CELF/MBNL pairs were derepressed more strongly than those containing exclusively distal pairs, consistent with the local inhibition phenomenon observed in Figure 5D. Together, these data indicate that the regulatory rules that govern CELF1 function in heart development are similar to those observed in the transgenic mouse model.

### No evidence for direct antagonism of binding for CELF and MBNL proteins

To help distinguish between the “local inhibition of binding” and “local inhibition of function” models introduced above, we performed a variant of our RNA Bind-n-Seq (RBNS) assay (Lambert et al. 2014), in which recombinant, tagged CELF1 protein was incubated with a pool of random RNA oligonucleotides (of length 40 nt), in the absence of MBNL1 or in the presence of varying concentrations of recombinant MBNL1 protein. This experiment can be thought of as mimicking the effects of developmental induction of MBNL1 on CELF1's interaction with RNA. Following pulldown of CELF1 using the affinity tag, bound RNA was eluted and used to prepare libraries for high-throughput sequencing (Methods). The calculation of motif enrichment (called RBNS *R*-values) in the CELF1 pulldown libraries relative to a control library prepared from input RNA enables assessment of sequence-specific RNA affinity.

By analyzing all RNA 6mers, we observed a strong preference for GU- and UGU-containing motifs such as UGUUGU in all pulldown libraries, consistent with previous studies (Faustino and Cooper 2005; Marquis et al. 2006; Lambert et al. 2014). By comparing data obtained with relatively high MBNL1 concentration (e.g., 250 nM, a concentration at which MBNL1 exhibits strong sequence-specific binding in this assay) to 0 MBNL concentration, we observed no effect of the presence of MBNL1 protein on the absolute or relative affinity of CELF1 for different RNA motifs (Fig. 5F), nor any effect of the presence of MBNL1 motifs in the oligonucleotide. These observations were consistent across a range of additional MBNL1 concentrations, from 64 nM to 1 μM (Supplemental Fig. S8). This result was not entirely unexpected, as CELFs and MBNLs have distinct sequence specificities, with CELF1 favoring GU- and UGU-containing motifs (Faustino and Cooper 2005; Marquis et al. 2006; Lambert et al. 2014), and MBNL1 favoring motifs containing UGC and/or GCU (Goers et al. 2010; Lambert et al. 2014). These data suggest that binding of these two proteins to RNA exhibits neither synergism nor antagonism, instead occurring independently. Of course, the usual caveats regarding the experimental setup and presence of an affinity tag on CELF1 apply, and the in vivo situation might be different. This conclusion provides no support for local inhibition of binding but is consistent with the local inhibition of function model.

## Discussion

The relationship between CELF and MBNL proteins is important both in the development and context of neuromuscular disease, particularly DM. Throughout mouse heart development, CELF protein levels decrease and MBNL levels increase (Kalsotra et al. 2008); in DM, MBNL proteins are sequestered by expanded CUG or CCUG repeats, and CELF proteins are stabilized via hyperphosphorylation by PKC and derepressed as a result of reduced miRNA expression (Timchenko et al. 2004; Kuyumcu-Martinez et al. 2007; Kalsotra et al. 2010). The nature of the relationship between these RBPs in normal physiology and development has been a long-standing question in the field.

**Figure 6.**
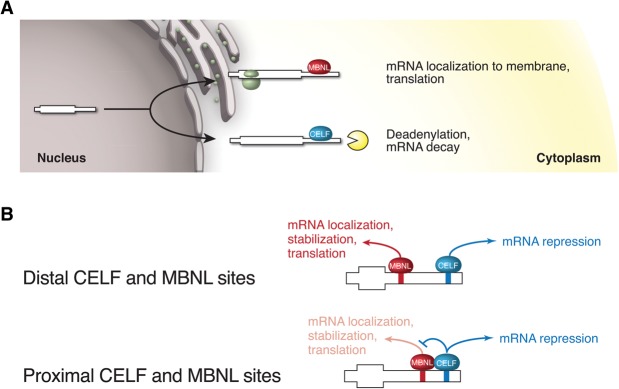
Functional antagonism model of effects of CELF and MBNL proteins on mRNA fates. (*A*) Summary of known nonsplicing activities: CELF1 binding to 3′ UTRs promotes mRNA deadenylation and decay (Vlasova and Bohjanen 2008), while MBNL1 binding to 3′ UTRs promotes localization to membrane compartments (Wang et al. 2012). (*B*) Binding of both proteins to the same mRNA is expected to result in a functional tug-of-war. For mRNAs containing distal MBNL and CELF binding sites, MBNL promotes targeting of the mRNA for localization and stabilization, while CELF binding promotes decay. For mRNAs containing proximal MBNL and CELF binding sites, CELF1 may directly antagonize MBNL1 function, e.g., by preventing recruitment of complexes associated with localization or inhibiting their function.

It has recently been established that many genes shift toward greater expression of longer 3′ UTR isoforms during differentiation of muscle cells and likely other cell types (Ji et al. 2009). We observed a strong pattern of antagonistic regulation of mRNA levels by CELF and MBNLs associated with binding to 3′ UTR regions. It is known that CELF1 can recruit deadenylases to 3′ UTRs and destabilize mRNAs (Vlasova et al. 2008), and recently we uncovered a global role for MBNLs in localizing mRNAs to membrane destinations for localized translation (Wang et al. 2012). These observations suggest a model in which MBNL and CELF proteins specify different cellular outcomes for mRNAs and compete with one another to determine the localization/stabilization or destabilization of specific mRNAs that contain binding motifs for both factors (Fig. 6A). Changes in localization often impact mRNA stability (Walters and Parker 2014). Our data, e.g., the pattern shown in Figure 5B, can be most simply explained if messages tend to have a basal decay rate in the cytoplasm, which is generally accelerated by *CELF1* induction and specifically enhanced by direct CELF1 binding, but have a lower decay rate when localized to the membrane compartment. Under this model, the localizations and half-lives of more than 1000 messages bound by both factors are determined by the outcome of a competition between the activities of MBNL and CELF proteins.

Down-regulation of the *CLCN1* gene, which encodes the major voltage-gated chloride channel that controls the membrane excitability of skeletal muscle, causes the myotonia that is characteristic of DM (Charlet et al. 2002; Mankodi et al. 2002). This down-regulation is thought to result primarily from aberrant inclusion of exon 7a, due to loss of *MBNL1* activity, which produces a nonfunctional *CLCN1* mRNA (Wheeler et al. 2007). Here, we showed that *Clcn1* mRNA levels decreased monotonically following *CELF1* induction in both muscle and heart and that CELF1 binds to the *Clcn1* 3′ UTR (Fig. 3C). These observations suggest that CELF1 may down-regulate the stability of *Clcn1* mRNA, independently of splicing regulation. Therefore, the up-regulation of CELF activity that often accompanies MBNL sequestration in DM skeletal muscle may destabilize the *CLCN1* mRNA and exacerbate myotonia symptoms.

Other messages bound by both factors whose expression responded to *CELF1* induction and whose localization changed in response to *Mbnl1* depletion (Results) included *Kcnj2*, a cardiac inward rectifier potassium channel whose mutation is associated with a syndrome that involves cardiac arrhythmia (Andelfinger et al. 2002), and the sine oculis binding protein homolog *Sobp*, mutations of which have been linked to intellectual disability (Birk et al. 2010). Other messages with this pattern of binding and localization included *Cpe*, which is involved in insulin processing, and *Igfbp5*, which regulates insulin-responsive growth factor. Thus, genes that encode competing mRNA targets of CELFs and MBNLs may contribute to symptoms such as cardiac arrhythmia, mental retardation, and insulin resistance, which are observed in DM.

The distance between CELF and MBNL sites on a message appears to play an important role in the outcome of competition between these factors: Close spacing of CELF1 sites near MBNL sites (less than ∼50 nt apart) appears to override the protective effect normally conferred by MBNL1 binding (Figs. 5D, 6B), either by inhibition of binding or of activity. Our in vitro binding data (Fig. 5F) did not detect evidence of a cooperative or antagonistic relationship between binding of CELF1 and MBNL1, suggesting that antagonism occurs at the functional level. For example, CELF binding may inhibit the recruitment or activity of MBNL-dependent complexes involved in mRNA localization or stabilization. Functional antagonism between RBPs that bind to the same mRNAs is well known; e.g., PTB can repress U1 snRNP's ability to promote spliceosome formation without inhibiting its binding to the 5′ splice site (Izquierdo et al. 2005; Sharma et al. 2005), and SRRM4 (also known as NSR100) can overcome the repressive effects of PTBP1 without generally interfering with its binding (Raj et al. 2014). The functional antagonism between MBNL and CELF proteins uncovered here is likely to contribute to the robustness of developmental changes in mRNA stability and localization, particularly for mRNAs bound by both factors (Fig. 5E). These mRNAs may be particularly susceptible to misregulation in DM, perhaps contributing to pathogenesis.

We identified several dozen exons exhibiting antagonistic splicing regulation, many of which are developmentally regulated. These exons tend to preserve the reading frame and to reside in genes affecting functions involved in development and cell differentiation (see Results). Human homologs of this set of antagonistically regulated exons may be misregulated in DM to a particularly strong extent, as increased levels of CELF proteins would exacerbate splicing changes that result from MBNL sequestration. The hypothesis that CELF binding to exons tends to repress splicing, suggested by genome-wide analyses, was confirmed in a splicing reporter system, establishing a rule to predict CELF-dependent splicing regulation.

CELF1 function intersects with RNA processing in a different way in its impact on the stability of alternative mRNA isoforms that differ in their 3′ UTRs. We observed a large set of genes with developmentally regulated ALEs and tandem UTR isoforms that differentially respond to CELF induction (Fig. 4). Regulation of some of these isoforms by CELF1 may impact encoded protein functions by changing the abundance of protein isoforms differing in their C termini, while regulation of others may alter mRNA localization or translation or other UTR-dependent properties during development and other physiological situations in which CELF activity changes.

## Methods

### Heart inducible *CELF2* mouse

N-terminal FLAG-tagged human CELF2 was expressed from the transgene previously used for CELF1 (Koshelev et al. 2010). *TRECUGBP2* transgenic mice were generated by standard techniques and maintained on an FVB background. All reported *TRECUGBP2*/*MHCrtTA* bitransgenic mice were the F1 progeny from *TRECUGBP2* × *MHCrtTA* (FVB/N-Tg(*Myh6*-*rtTA*)1Jam) matings. *TRECUGBP2/MHCrtTA* bitransgenic mice induced using doxycycline did not show evidence of ECG, echocardiography abnormalities, or abnormal heart size or morphology after 8 wk of induced expression of *CELF2*.

### RNA-seq library preparation and sequencing

*CELF1* and *CELF2* were induced in the heart and muscle by feeding mice 2 g/kg doxycycline for 12, 24, 72 h, or 7 d in the relevant transgenic animal. For *CELF1* induction in the heart, mice with myosin heavy chain, promoter-driven, reverse tetracycline transactivator (*rtTA*) and Tet-inducible, N-terminal FLAG-tagged human CELF1 LYLQ isoform were used (data are provided under GEO accession GSE56185) (Giudice et al. 2014). For *CELF2* induction in the heart, mice with the myosin heavy chain promoter-driven *rtTA* and Tet-inducible human *CELF2* were used. Hearts were harvested, atria removed, and ventricles frozen in liquid nitrogen. For *CELF1* induction in muscle, mice with the rat myosin light chain 1/3 promoter/enhancer driving *rtTA* and Tet-inducible, N-terminal FLAG-tagged human *CELF1* LYLQ isoform were used. The left and right gastrocnemius were isolated and frozen in liquid nitrogen. Control experiments for all time courses used mice with *rtTA* cassettes but lacking the Tet-inducible *CELF* cassettes; control mice were fed 2 g/kg doxycycline for 72 h before tissue harvest. Total RNA was isolated in all cases using TRIzol (Invitrogen), followed by RNeasy column with DNase treatment (Qiagen). Poly(A)^+^ RNA was prepared using Oligo dT dynabeads (Invitrogen) and prepared for paired-end RNA-seq (36–40 nt on Illumina GAII).

### CLIP-seq library preparation and sequencing

CLIP was performed using 254-nm UV irradiation as previously described (Wang et al. 2009b), using heart tissue or muscle tissue of 16-wk-old mice or cultured C2C12 mouse myoblasts. Tissue was ground to a powder using a liquid nitrogen–cooled mortar and pestle prior to UV irradiation. The dry powder was placed into a 10-cm^2^ tissue culture dish, sitting on ice, and crosslinked 3 × 400 mJ/cm^2^. In between each round of cross-linking, the dish was shaken from side to side to redistribute the tissue powder and provide maximum opportunity for all tissue particles to be cross-linked. The tissue was then lysed in RIPA buffer (50 mM Tris-HCl at pH 7.4, 150 mM NaCl, 0.1% sodium deoxycholate, 1% NP-40, 0.5% SDS). The lysate was treated with DNase and RNase I_f_ (NEB) for 10 min at 37° C, with dilutions of 1:10,000 and 1:50,000 providing optimal RNA fragment lengths for downstream purification. Immunoprecipitation was performed using the 3B1 antibody clone against CELF1 (Millipore) and protein A beads (Invitrogen). The beads were washed twice with RIPA and twice with RIPA containing 1 M NaCl. The 3′ adapter was preadenylated with ImpA (Hafner et al. 2008) and ligated while the RNA–protein complexes were on beads using T4 RNA ligase (Rnl2 truncation) in the absence of ATP (NEB). The complexes were run on SDS-PAGE gel, transferred to nitrocellulose, and isolated from membrane as previously described (Wang et al. 2009b). Further details are provided in the Supplemental Material.

### RNA-seq and CLIP-seq read mapping, gene expression estimation

All read mapping was performed using Bowtie/TopHat (Langmead et al. 2009; Trapnell et al. 2009), mapping to mm9. Only uniquely mapping reads were used. CLIP reads were collapsed to remove identical sequences; adapter sequences were removed; and processed reads were then mapped separately for each CLIP read length. To estimate gene expression levels, the number of reads mapping to each kilobase of constitutive coding sequence of RefSeq/Locuslink genes was counted and divided by the number of reads (in millions) mapping uniquely to nonribosomal and nonmitochondrial sequence to obtain RPKM values. For purposes of the analyses performed in Figure 4E, gene expression values in the heart development time course were normalized as previously described (Robinson and Oshlack 2010), using parameters *M* = 0.3 and *A* = 0.2.

### Estimation of isoform frequencies, calculation of MZ score

MISO (version 0.4.8) (Katz et al. 2010) was used to estimate isoform frequencies for splicing events and alternative 3′ UTR events, using a minimum of 20 reads per event and the parameters of burn_in=500, lag=10, num_iters=5000, and num_chains=6. To identify splicing events that change monotonically over time, we ordered samples chronologically, and for each event compared all pairs of samples from different time points, tallying the number of comparisons representing significant increase or significant decrease in Ψ (at BF > 5). We calculated a quantity called δ, the number of significant positive ΔΨ values (increases over time) minus the number of significant negative ΔΨ values. To assess statistical significance, we recalculated δ after randomly permuting the sample labels. Repeating this process 100 times, we generated a null distribution and derived the “monotonicity *Z*-score” (MZ = (δ − μ)/σ), where μ and σ are the mean and standard deviation of the null distribution, respectively.

### Analysis of antagonistically regulated splicing events

Splicing events regulated in response to each perturbation (*CELF* overexpression, *Mbnl1* KO, or heart development) were enumerated, and the number of events regulated among each pair of perturbations was counted (this was the “observed” overlap) (Fig. 1G,H). To compute the “expected” overlap, we assumed independence; e.g., the fraction of events regulated in both perturbations equals the fraction of events regulated in the first perturbation multiplied by the fraction of events regulated in the second perturbation. Significance of the bias in direction of regulation (Fig. 1H) was assessed by binomial test, assuming a null hypothesis frequency of 0.5.

### Analysis of CLIP clusters and correlation across the transcriptome

Analysis of CLIP data was performed as described in the Supplemental Material (Fig. 2B,C,E; Supplemental Fig. S3B,C).

### Conservation analysis for cassette exons and 3′ UTRs

Conservation (30-way phastCons, UCSC Genome Browser) of cassette exons with CLIP clusters within 1 kb of each splice site or within the exon itself was compared to a control set of cassette exons found in similarly expressed genes (Figs. 2D, 3B). Conservation (30-way phastCons) of 3′ UTRs with CLIP clusters <1 kb upstream of and <500 bases downstream from the constitutive transcript ends was assessed and compared to a control set of 3′ UTRs with similar length, in similarly expressed genes. For these analyses, CLIP clusters and gene expression values from heart were used.

### Analysis of gene expression changes as a function of CELF CLIP clusters and/or miRNA seed matches

Genes were grouped by the number of CELF CLIP clusters found in 3′ UTRs (Fig. 3D) or miRNA seed matches (Supplemental Fig. S5A), and the mean log_2_(fold-change) in expression level in each group was computed, relative to genes with no CLIP clusters or miRNA seeds, respectively (Fig. 3D; Supplemental Fig. S5A). Significance was assessed by rank-sum test. The abundance of miRNAs in heart (Supplemental Fig. S5B) was derived from Li et al. (2013).

### Analysis of binding targets shared by CELF1 and MBNL1

Genes were binned by expression level in mouse myoblast (Wang et al. 2012), and within each bin, the number of genes with 3′ UTRs containing CELF1 CLIP clusters only, MBNL1 CLIP clusters only, or clusters for both proteins was counted (“observed” number). The “expected” number was computed by assuming independence. The approximately threefold observed/expected value was obtained by taking the mean observed/expected value across all gene expression bins. In Figure 5A, CLIP data for CELF1 were derived from muscle; data for MBNL1 were derived from myoblasts.

### Analysis of CELF1 and MBNL1 binding locations within 3′ UTRs

To precisely assess the distance between CELF1 and MBNL1 binding locations within 3′ UTRs, we searched for known CELF1 and MBNL1 binding motifs within each CLIP cluster for each protein (Fig. 5C–E; Supplemental Fig. S7A,B). The motifs were derived from Bind-n-Seq data (Lambert et al. 2014). For MBNL1, the motifs were GCTT, CGCT, TGCT, GCGC, CCGC, CTGC, GCTA, ACGC, CGCA, AGCT, TTGC, and CAGC. For CELF1, the motifs were TGTT, ATGT, TTGT, TGTC, GTGT, TGTA, GTTT, TGTG, GTCT, and TTTT. If none of these motifs was found within the cluster, the binding location was estimated to be the center of the CLIP cluster. The closest distance between each CELF1 and MBNL1 motif derived from its respective cluster was recorded and compared with randomly assigned clusters (Fig. 5C) or clusters whose identity was shuffled (Supplemental Fig. S7A). Tests for significant differences in Figure 5C and Supplemental Figure S7A were performed by modified KS test, where cumulative distributions were visually inspected to confirm a true shift in median values.

### Analysis of alternative 3′ UTR isoforms

The abundance of alternative 3′ UTR isoforms by MISO was performed in “multi-isoform” mode (Fig. 4B–E; Supplemental Fig. S6). However, analysis was restricted to those events with exactly two isoforms whose regulation is significant and monotonic (BF > 5, MZ > 1.6 for tandem UTRs, MZ > 1.5 for ALEs). To assess changes in gene expression, genes were binned by the number of CELF1 CLIP clusters in the “core” or “extension” regions (Fig. 4B) or in both ALEs (Fig. 4E), and the mean expression change following *CELF1* induction in each bin was compared to the mean expression change in the bin with zero CELF1 CLIP clusters. Significance was assessed by rank-sum test. To estimate the “potency” of each CLIP cluster, we used a linear model; for tandem UTRs, we considered a linear model in which log(expression change) is proportional to the number of core sites × potency of core site + number of extension sites × potency of extension × Ψ_extension_. For ALEs, we considered a model in which log(expression change) is proportional to the number of sites in ALE1 × potency × Ψ_ALE1_ + number of sites in ALE2 × potency × Ψ_ALE2_. In both cases, coefficients were estimated by least squares regression.

## Data access

The RNA-seq, CLIP-Seq, and RBNS sequencing data from this study have been submitted to the NCBI Gene Expression Omnibus (GEO; http://www.ncbi.nlm.nih.gov/geo/) under superseries accession number GSE61893.

## Supplementary Material

Supplemental Material
