# Supplementary material for: Antagonistic regulation of mRNA expression and splicing by CELF and MBNL proteins
Source: Genome Res. 2015 Jun;25(6):858–71. doi: 10.1101/gr.184390.114 (PMC4448682; doi:10.1101/gr.184390.114)
Supplement: Supplemental Material [file supp_25_6_858__index.html]

Antagonistic Regulation of mRNA Expression and Splicing by CELF and MBNL Proteins — Antagonistic regulation of mRNA expression and splicing by CELF and MBNL proteins — Antagonistic regulation of mRNA expression and splicing by CELF and MBNL proteins — Supplemental Material 

# Antagonistic regulation of mRNA expression and splicing by CELF and MBNL proteins

## Supplemental Material

**Files in this Data Supplement:**

- Supp Material & Figures.pdf
- Supp Table S1.xlsx
- Supp Table S2.xlsx
- Supp Table S3.xlsx
- Supp Table S4.xlsx
- Supp Table S5.xlsx
- Supp Table S6.xlsx
- Supp Table S7.xlsx
- Supp Table S8.xlsx
