# Supplementary material for: Antagonistic regulation of mRNA expression and splicing by CELF and MBNL proteins
Source: Genome Res. 2015 Jun;25(6):858–71. doi: 10.1101/gr.184390.114 (PMC4448682; doi:10.1101/gr.184390.114)
Supplement: Supplemental Material [file supp_gr.184390.114_SuppMaterial_Figures.pdf]

Extended Experimental Procedures

CLIP-seq library preparation and sequencing

CLIP was performed using 254 nm UV irradiation as previously described (Wang et al. 2009), using heart tissue or muscle tissue of 16-week-old mice or cultured C2C12 mouse myoblasts. Tissue was ground to a powder using liquid nitrogen-cooled mortar and pestle prior to UV irradiation. The dry powder was placed into a 10 cm^2 tissue culture dish, sitting on ice, and crosslinked 3 x 400 mJ/cm^2. In between each round of cross-linking, the dish was shaken from side to side, to redistribute the tissue powder and provide maximum opportunity for all tissue particles to be cross-linked. The tissue was then lysed in RIPA buffer (50 mM Tris-HCl pH 7.4, 150 mM NaCl, 0.1% sodium deoxycholate, 1% NP-40, 0.5% SDS). The lysate was treated with DNase and RNase I_f (NEB) for 10 minutes at 37 degrees, with dilutions of 1:10,000 and 1:50,000 providing optimal RNA fragment lengths for downstream purification. Immunoprecipitation was performed using the 3B1 antibody clone against CELF1 (Millipore) and protein A beads (Invitrogen). The beads were washed twice with RIPA and twice with RIPA containing 1M NaCl. The 3' adapter was pre-adenylated with ImpA (Hafner et al. 2008) and ligated while the RNA-protein complexes were on beads using T4 RNA ligase (Rnl2 truncation) in the absence of ATP (NEB). The complexes were run on SDS-PAGE gel, transferred to nitrocellulose, and isolated from membrane as previously described (Wang et al. 2009). The protein was digested by proteinase K, and RNA was precipitated, and ligated to the 5' adapter using T4 RNA ligase (NEB). Dinucleotide barcode sequences were used at the 3' end of the 5' adapter, to allow for censoring of CLIP read PCR duplications. Ligated products were resolved by electrophoresis on TBE-urea polyacrylamide gels, isolated, and subjected to RT-PCR using Superscript III enzyme (Invitrogen) and Phusion polymerase (NEB). PCR primers contained indexes for sequence multiplexing, but for these experiments no samples were multiplexed. Adapter and primer sequences (5' to 3') were as follows:

3' Adapter	/5Phos/TGGAATTCTCGGGTGCCAAGG/3ddC/
5' Adapter	/5AmMC6/GTTCAGAGTTCTArCrArGrUrCrCrGrArCrGrArUrCrNrN
RT primer	AATGATACGGCGACCACCGACAGGTTCAGAGTTCTACAGTCCGA
Forward PCR primer	AATGATACGGCGACCACCGAGATCTACACGTTCAGAGTTCTACAGTCCGA
Rev. PCR primer 1	CAAGCAGAAGACGGCATACGAGATCGTGATGTGACTGGAGTTCCTTGGCACCCGAGAATTCCA
Rev. PCR primer 2	CAAGCAGAAGACGGCATACGAGATACATCGGTGACTGGAGTTCCTTGGCACCCGAGAATTCCA
Rev. PCR primer 3	CAAGCAGAAGACGGCATACGAGATGCCTAAGTGACTGGAGTTCCTTGGCACCCGAGAATTCCA
Rev. PCR primer 4	CAAGCAGAAGACGGCATACGAGATTGGTCAGTGACTGGAGTTCCTTGGCACCCGAGAATTCCA

RNA-seq and CLIP-seq read mapping, gene expression estimation

All read mapping was performed using Bowtie/Tophat (Langmead et al. 2009; Trapnell et al. 2009), mapping to mm9. Only uniquely mapping reads were used. CLIP reads were collapsed to remove identical sequences, adapter sequences were removed, and processed reads were then mapped separately for each CLIP read length. To estimate gene expression levels, the number of reads mapping to each kilobase of constitutive coding sequence of RefSeq/Locuslink genes was counted, and divided by the number of reads (in millions) mapping uniquely to non-ribosomal and non-mitochondrial sequence, to obtain RPKM values. For purposes of the analyses performed in Fig. 4E, gene expression values in the heart development time course were normalized as described (Robinson and Oshlack 2010), using parameters of M = 0.3 and A = 0.2.

Estimation of isoform frequencies, calculation of MZ score

MISO (version 0.4.8) was used to estimate isoform frequencies for splicing events and alternative 3' UTR events, using a minimum of 20 reads per event and parameters of burn_in=500, lag=10, num_iters=5000, and num_chains=6. To identify splicing events that change monotonically over time, we ordered samples chronologically, and for each event compared all pairs of samples from different time points, tallying the number of comparisons representing significant increase or significant decrease in Ψ (at BF > 5). We calculated a quantity called δ, the number of significant positive $\Delta\Psi$ values (increases over time) minus the number of significant negative $\Delta\Psi$ values. To assess statistical significance, we recalculated δ after randomly permuting the sample labels. Repeating this process 100 times, we generated a null distribution, and derived the

“monotonicity Z-score” (MZ = $(\delta - \mu) / \sigma$), where μ and σ are the mean and standard deviation of the null distribution, respectively.

Analysis of antagonistically regulated splicing events (Figs. 1G, 1H)

Splicing events regulated in response to each perturbation (CELF over-expression, Mbnl1 KO, or heart development) were enumerated, and the number of events regulated among each pair of perturbations was counted (this was the “observed” overlap). To compute the “expected” overlap, we assumed independence, e.g. the fraction of events regulated in both perturbations equals the fraction of events regulated in the first perturbation multiplied by the fraction of events regulated in the second perturbation. Significance of the bias in direction of regulation (Figure 1h) was assessed by binomial test, assuming a null hypothesis frequency of 0.5.

Correlation of CLIP binding density across the transcriptome (Fig. 2B)

CLIP read density was computed in 5 nt windows across 3' UTRs in the transcriptome for genes highly expressed in whole brain, heart, muscle, and C2C12 mouse myoblasts (>100 RPKM) exhibiting high CLIP coverage (>100 tags per UTR). Pearson correlation coefficients were computed for these densities between CLIP libraries performed in different samples and for different proteins.

Identification of CLIP clusters

CLIP tags were first collapsed to remove redundant sequences, and trimmed of adapters. These sequences were mapped to genome and a database of splice junctions using Bowtie. To identify CLIP clusters lying within genic regions, gene boundaries were first defined using RefSeq, Ensembl, and UCSC tables. For each window of 30 nucleotides covered by at least one CLIP tag, a test was performed to assess CLIP density in the window exceeded that which is predicted by a simple Poisson model which accounts for gene expression and pre-mRNA length (Yeo et al. 2009).

CLIP cluster motif analysis (Fig. 2C, Fig. S3B)

Pentamers occurring in CELF1 CLIP clusters from heart were counted and compared to those found in randomly selected clusters within the same 3' UTRs (Fig. 2C) or whole genes (Fig. S3B). This procedure was repeated 100 times to derive a Z-score for each motif, where the Z-score was defined as the number of standard deviations away from the mean. The 5 most highly enriched 5mers are highlighted.

Conservation analysis for cassette exons and 3' UTRs (Figs. 2D, 3B)

Conservation (30-way phastCons, UCSC genome browser) of cassette exons with CLIP clusters within 1 kilobase of each splice site or within the exon itself was compared to a control set of cassette exons found in similarly expressed genes. Conservation (30-way phastCons) of 3' UTRs with CLIP clusters less than 1 kilobase upstream and less than 500 bases downstream of constitutive transcript ends was compared to a control set of 3' UTRs with similar length, in similarly expressed genes. For these analyses, CLIP clusters and gene expression values from heart were used.

Crosslink-induced substitution analysis (Figs. 2E, S3C)

As in (Wang et al. 2012), p(CIS) was defined as the fraction of reads coverage a base supporting a base substitution differing from reference. Only positions with minimum read coverage above 10 were considered in this analysis. Information content of each base flanking frequently substituted cytosines was computed as 2 – $\sum_{[A,U,C,G]} f \log 2\ (f)$–, where f is the frequency of each base.

Supplemental References

Hafner M, Landgraf P, Ludwig J, Rice A, Ojo T, Lin C, Holoch D, Lim C, Tuschl T. 2008. Identification of microRNAs and other small regulatory RNAs using cDNA library sequencing. *Methods* **44**(1): 3-12.

Langmead B, Trapnell C, Pop M, Salzberg SL. 2009. Ultrafast and memory-efficient alignment of short DNA sequences to the human genome. *Genome Biol* **10**(3): R25.

Robinson MD, Oshlack A. 2010. A scaling normalization method for differential expression analysis of RNA-seq data. *Genome Biol* **11**(3): R25.

Trapnell C, Pachter L, Salzberg SL. 2009. TopHat: discovering splice junctions with RNA-Seq. *Bioinformatics* **25**(9): 1105-1111.

Wang ET, Cody NA, Jog S, Biancolella M, Wang TT, Treacy DJ, Luo S, Schroth GP, Housman DE, Reddy S et al. 2012. Transcriptome-wide regulation of pre-mRNA splicing and mRNA localization by muscleblind proteins. *Cell* **150**(4): 710-724.

Wang Z, Tollervey J, Briese M, Turner D, Ule J. 2009. CLIP: construction of cDNA libraries for high-throughput sequencing from RNAs cross-linked to proteins in vivo. *Methods* **48**(3): 287-293.

Yeo GW, Coufal NG, Liang TY, Peng GE, Fu XD, Gage FH. 2009. An RNA code for the FOX2 splicing regulator revealed by mapping RNA-protein interactions in stem cells. *Nat Struct Mol Biol* **16**(2): 130-137.

Supplementary Table and Figure Summary

Table S1. Mice used for RNA-seq. Related to Figure 1. (Excel file)

Table S2. All splicing events detected in *CELF1* and *CELF2* OE time courses. Related to Figure 1. (Excel file)

Table S3. Splicing events regulated by both *CELF* and *Mbnl*, along with information about regulation during heart development. Related to Figure 1. (Excel file)

Table S4. Gene Ontology analysis of genes containing exons responsive to both *CELF1* OE and *Mbnl1* depletion. Related to Figure 1. (Excel file)

Table S5. Gene Ontology categories for genes with 3' UTR CELF1 binding in heart, muscle, or C2C12. Related to Figure 2. (Excel file)

Table S6. Gene expression changes in response to *CELF* induction. Related to Figure 3. (Excel file)

Table S7. CELF1 and MBNL1 CLIP cluster locations within 3' UTRs. Related to Figure 5 (Excel file)

Table S8. Gene Ontology categories for genes with 3' UTR CELF1 binding in muscle and 3' UTR MBNL1 binding in C2C12 myoblasts. Related to Figure 5. (Excel file)

Figure S1. Gene expression levels as estimated by RNA-seq, for genes induced during *CELF1* or *CELF2* time courses. Related to Figure 1.

Figure S2. Method for determining monotonicity of splicing regulation. Related to Experimental Procedures and Figure 1.

Figure S3. CELF1 CLIP-seq in heart, muscle, and myoblasts. Related to Figure 2.

Figure S4. CELF1 binding is moderately associated with splicing regulation. Related to Figure 2.

Figure S5. ***CELF1* induction in heart leads to de-repression of miRNA targets.** Related to Figure 3.

Figure S6. Alternative last exon abundance as a function of the difference in number of CELF1 CLIP clusters between each 3' UTR. Related to Figure 4.

Figure S7. CELF1 and MBNL1 binding sites are closer than expected. Related to Figure 5.

Figure S8. MBNL1 presence does not affect in vitro binding of CELF1 to RNA. Related to Figure 5.

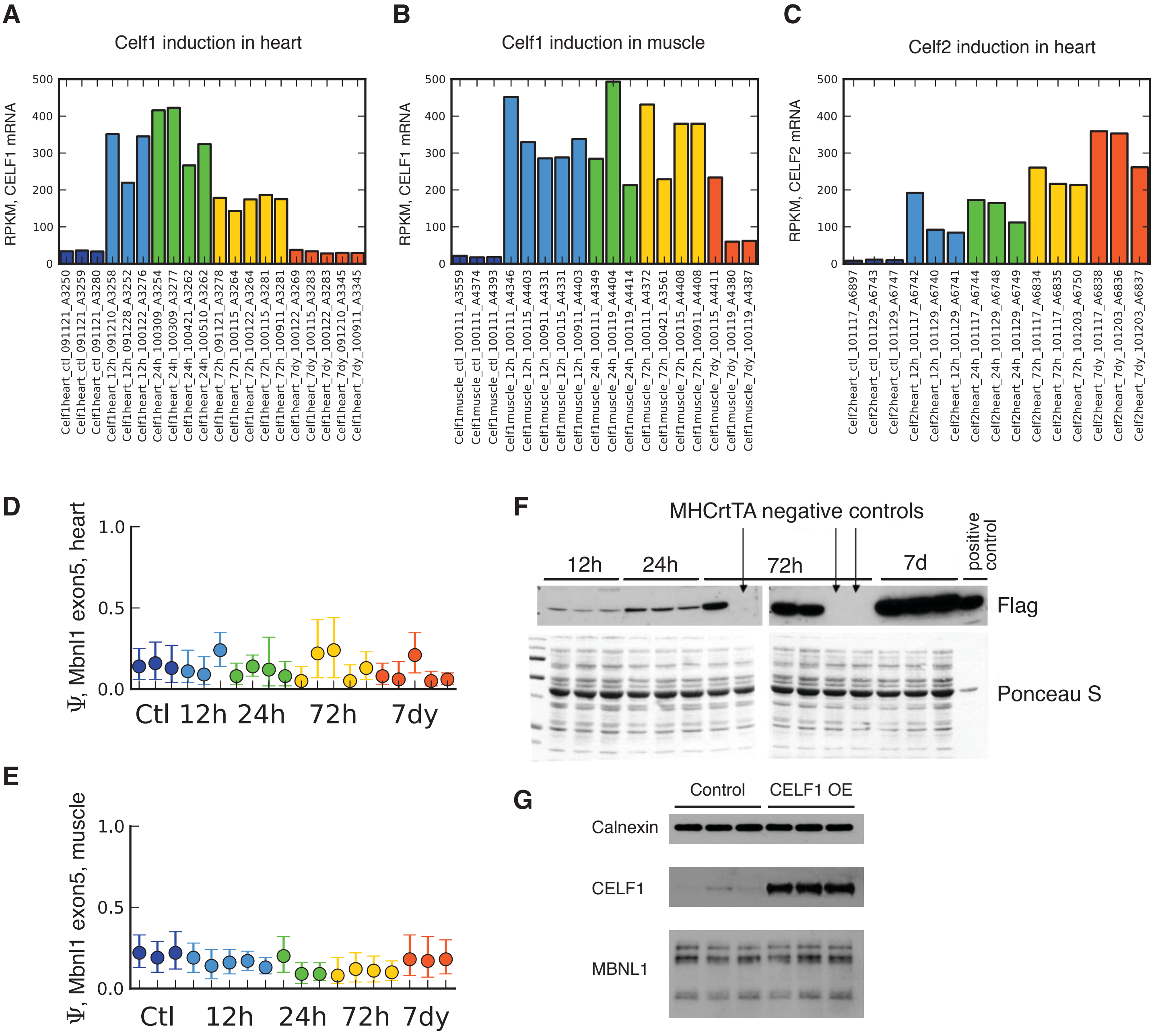

Figure S1. Gene expression levels as estimated by RNA-seq, for genes induced during *CELF1* or *CELF2* time courses. A) *CELF1* mRNA expression in heart, at various time points following *CELF1* induction in heart. B) *CELF1* mRNA expression in muscle, at various time points following *CELF1* induction in muscle. C) *CELF2* mRNA expression in heart, at various time points following *CELF2* induction in heart. D) *Mbnl1* exon 5 Ψ, at various time points following *CELF1* induction in heart. E) *Mbnl1* exon 5 Ψ, at various time points following *CELF1* induction in muscle. F) CELF2-Flag fusion protein expression by Western (HRP-conjugated anti-Flag antibody, Sigma A8592), and total protein stain by Ponceau S for each heart sample. 90 ug of total protein from the apex of each heart was loaded. G) CELF1 (mouse monoclonal 3B1 anti-CELF1 antibody, Millipore) and MBNL1 (mouse monoclonal MB1a 4A8 anti-MBNL1 antibody, Glenn Morris - CIND) protein expression by Western analysis, with Calnexin loading control (rabbit polyclonal anti-Calnexin antibody, Abcam ab22595) in response to *CELF1* over-expression in C2C12 myoblasts. MBNL1 protein levels remain unperturbed following *CELF1* OE.

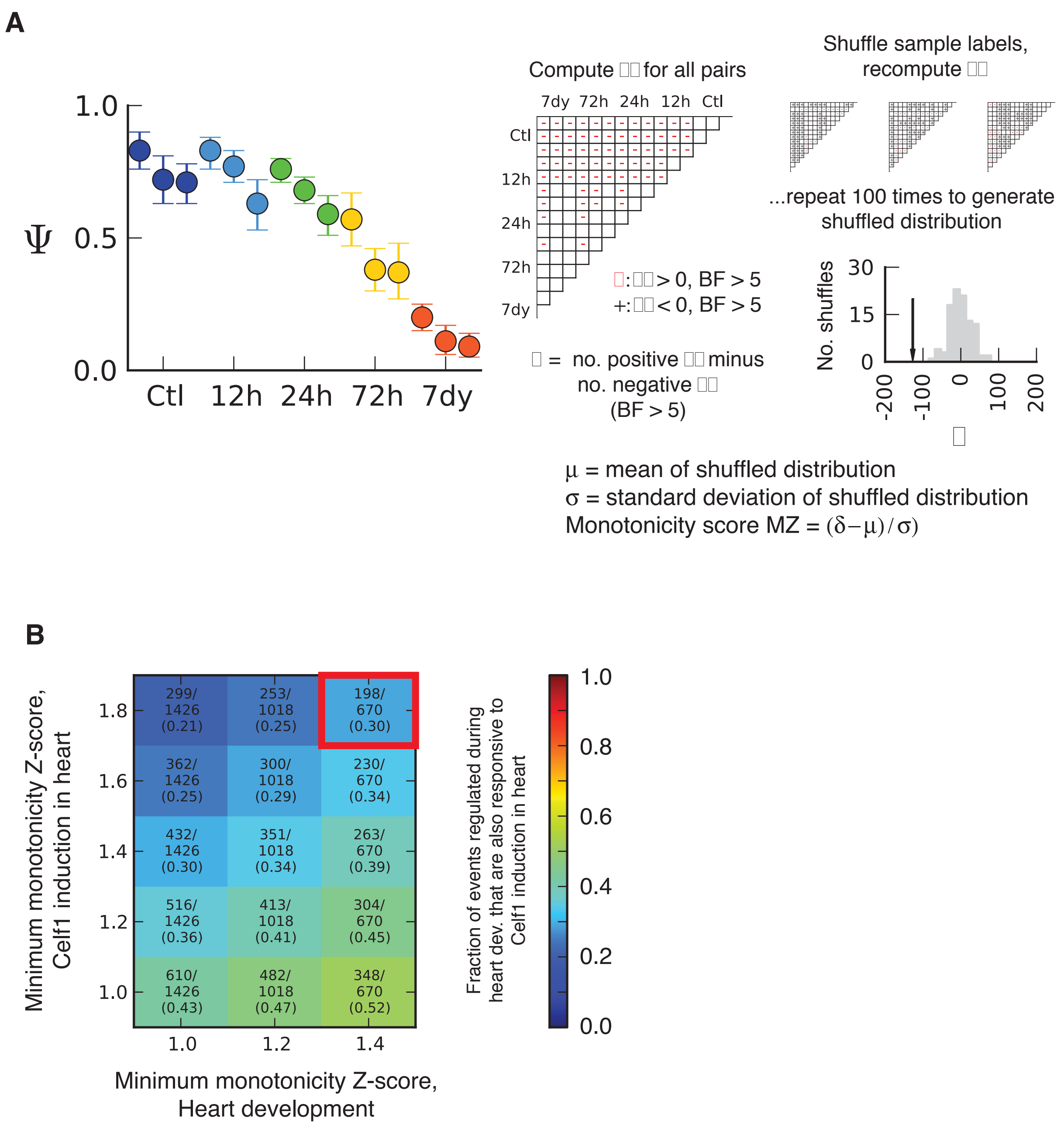

Figure S2. Method for determining monotonicity of splicing regulation. A) Method for determining monotonicity Z-score (MZ) of splicing changes. B) The number of splicing events regulated during heart development that are also responsive to *CELF1* induction were counted, at various MZ thresholds. The fraction of events at each threshold is also listed and represented by color in the heatmap. The combination of MZ thresholds used to create the scatter plot shown in Figure 1e is boxed in red.

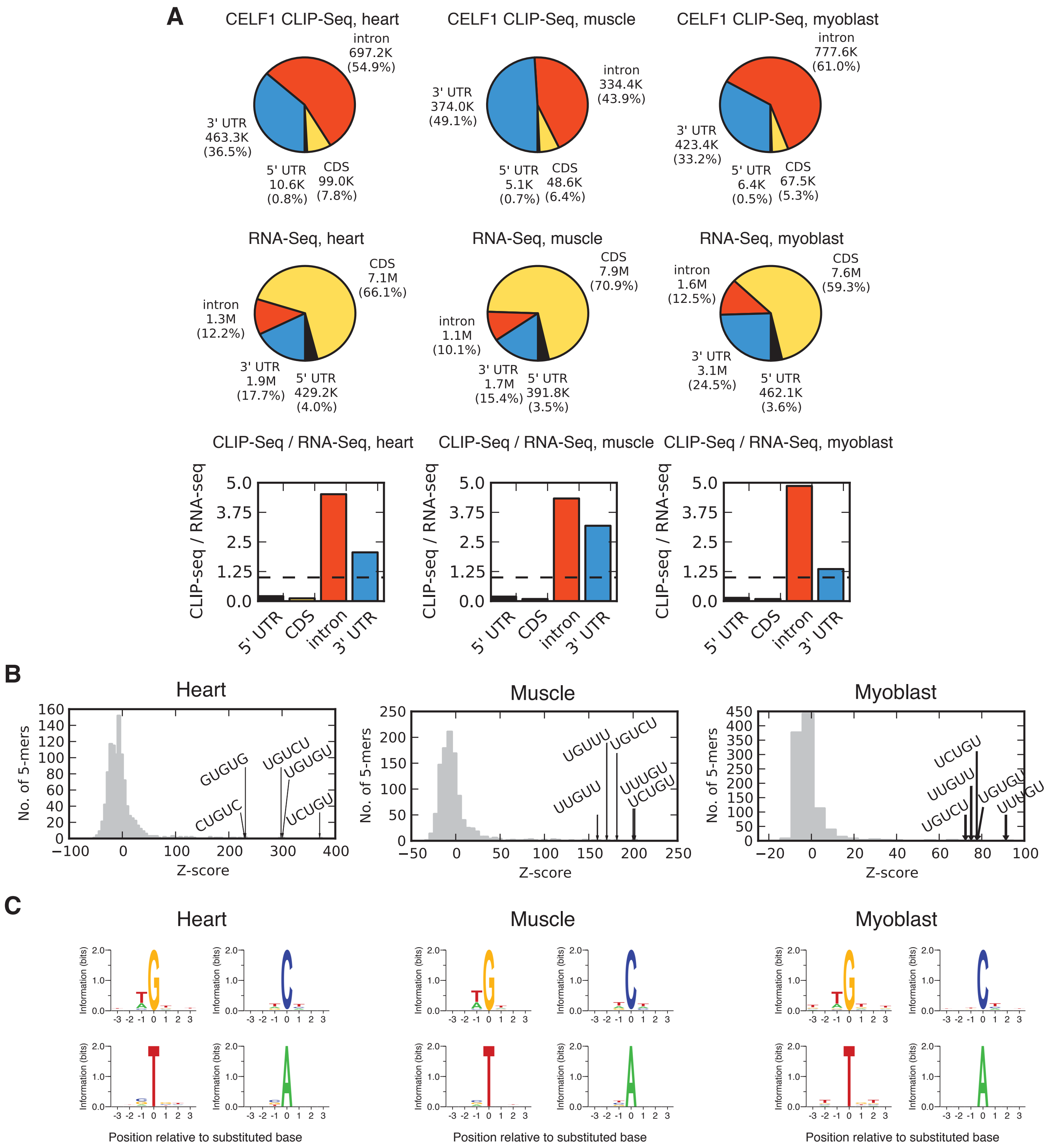

Figure S3. CELF1 CLIP-seq in heart, muscle, and myoblasts. A) The proportion of reads mapping to 5' UTRs, coding sequence, introns, and 3' UTRs is shown as pie charts for CLIP-seq and RNA-seq from heart, muscle, and myoblasts. The ratio between these values is shown as bar plots, illustrating enrichment for introns and 3' UTRs in CLIP reads relative to RNA-seq reads. B) Motif enrichment Z-scores for all pentamers occurring in CELF1 CLIP-seq clusters, relative to randomly selected genic regions, are shown for CELF1 CLIP clusters identified in heart, muscle, and myoblasts. C) Information content of nucleotides adjacent to sites with >30% crosslink-induced substitutions is illustrated.

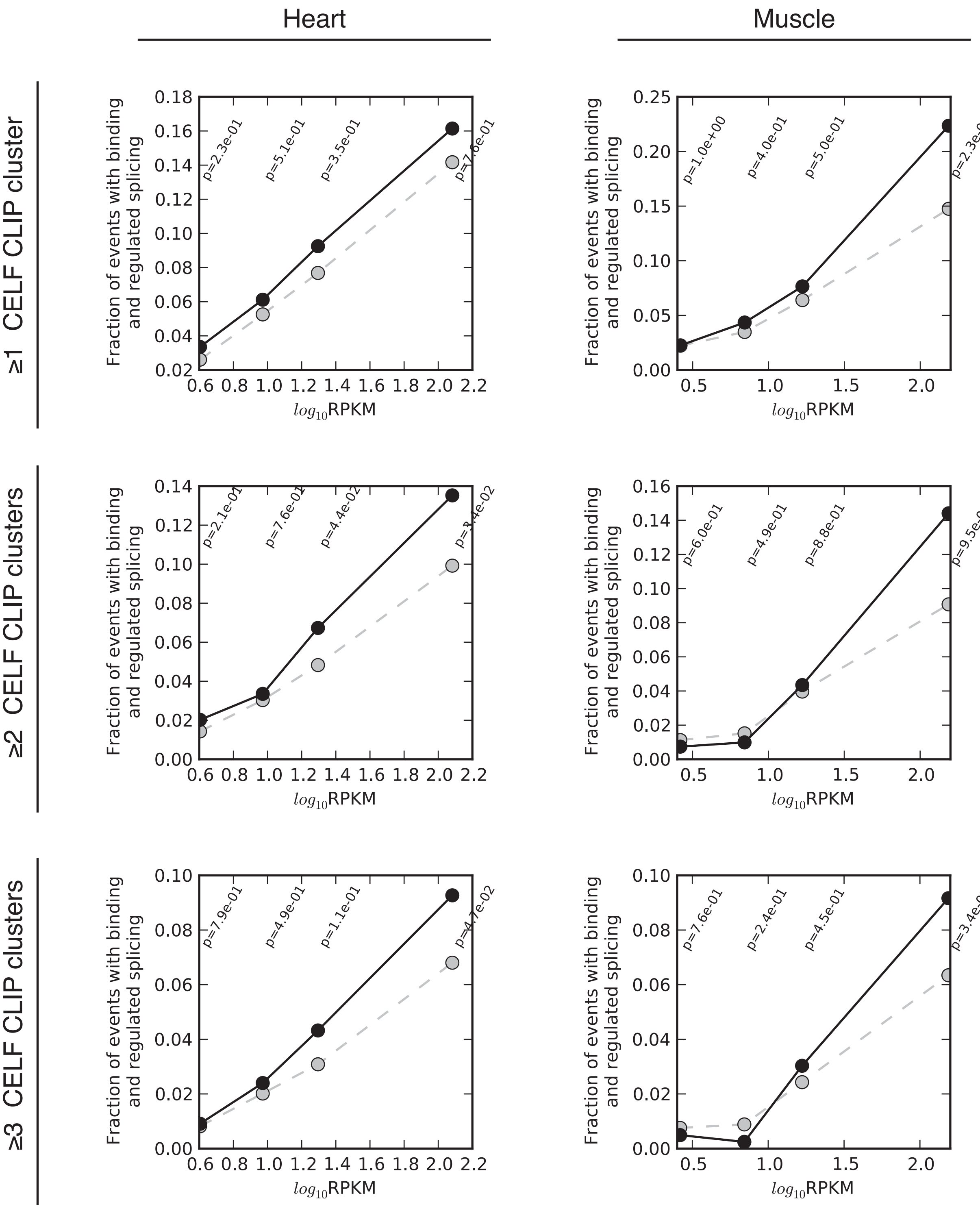

Figure S4. CELF1 binding is moderately associated with splicing regulation. Splicing events were binned by gene expression level (RPKM), and the observed fraction of splicing events with both binding and splicing regulation was plotted in black dots. Splicing regulation was defined using a minimum MZ of 2, and binding between 1 kb upstream of the 3' splice site and 1 kb downstream of the 5' splice site was counted. The expected fraction of events with both binding and regulation was estimated by assuming independence, and plotted in gray dots. A Fisher's exact test was performed to assess whether the observed fractions were different than expected, at each gene expression level bin. The analysis was performed separately in heart and muscle, for splicing events with at least 1, 2, or 3 CELF1 CLIP clusters. There was a modest (~10-40%) enrichment for splicing regulation of bound splicing events.

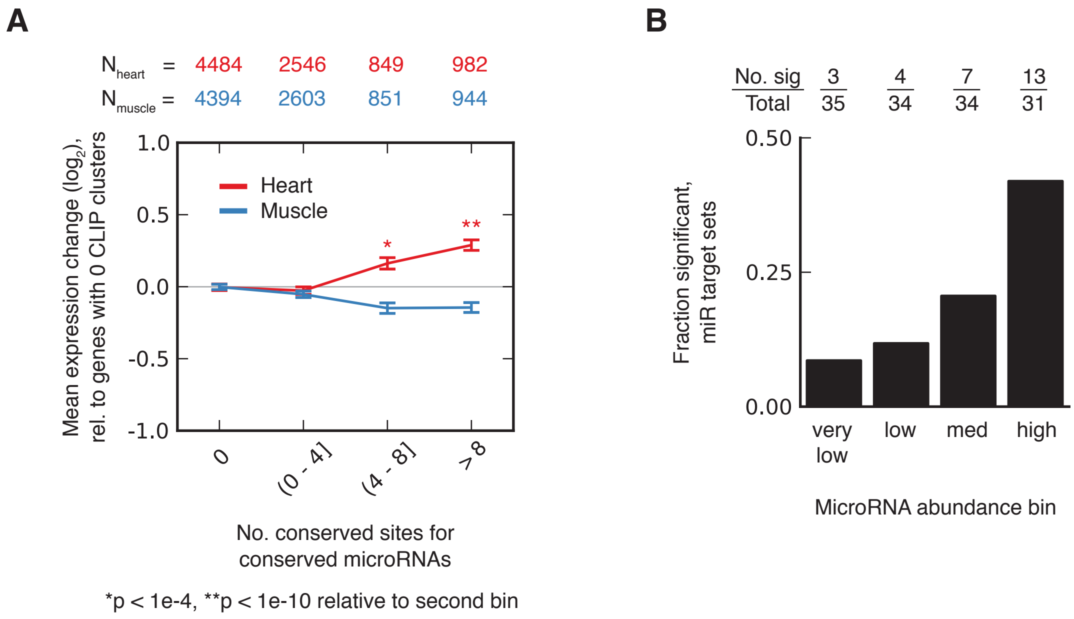

Figure S5. ***CELF1*** **induction in heart leads to de-repression of microRNA targets.** A) Mean log expression change following *CELF1* induction (7 d over control) for transcripts grouped by number of number of conserved target sites to conserved miRNAs in their 3' UTRs. Transcripts with greater numbers of conserved miRNA seed matches are de-repressed in heart but not in muscle. Significance was assessed by rank-sum test, where each microRNA seed bin was compared to the zero microRNA seed bin. B) The expression changes of miRNA target gene sets was analyzed, and the number of sets with significant derepression (P < 1e-4 by rank-sum test) is listed above for each of four cohorts of miRNAs, grouped by miRNA abundance in heart, from lowest to highest. Target sets corresponding to highly expressed miRNAs are more often derepressed.

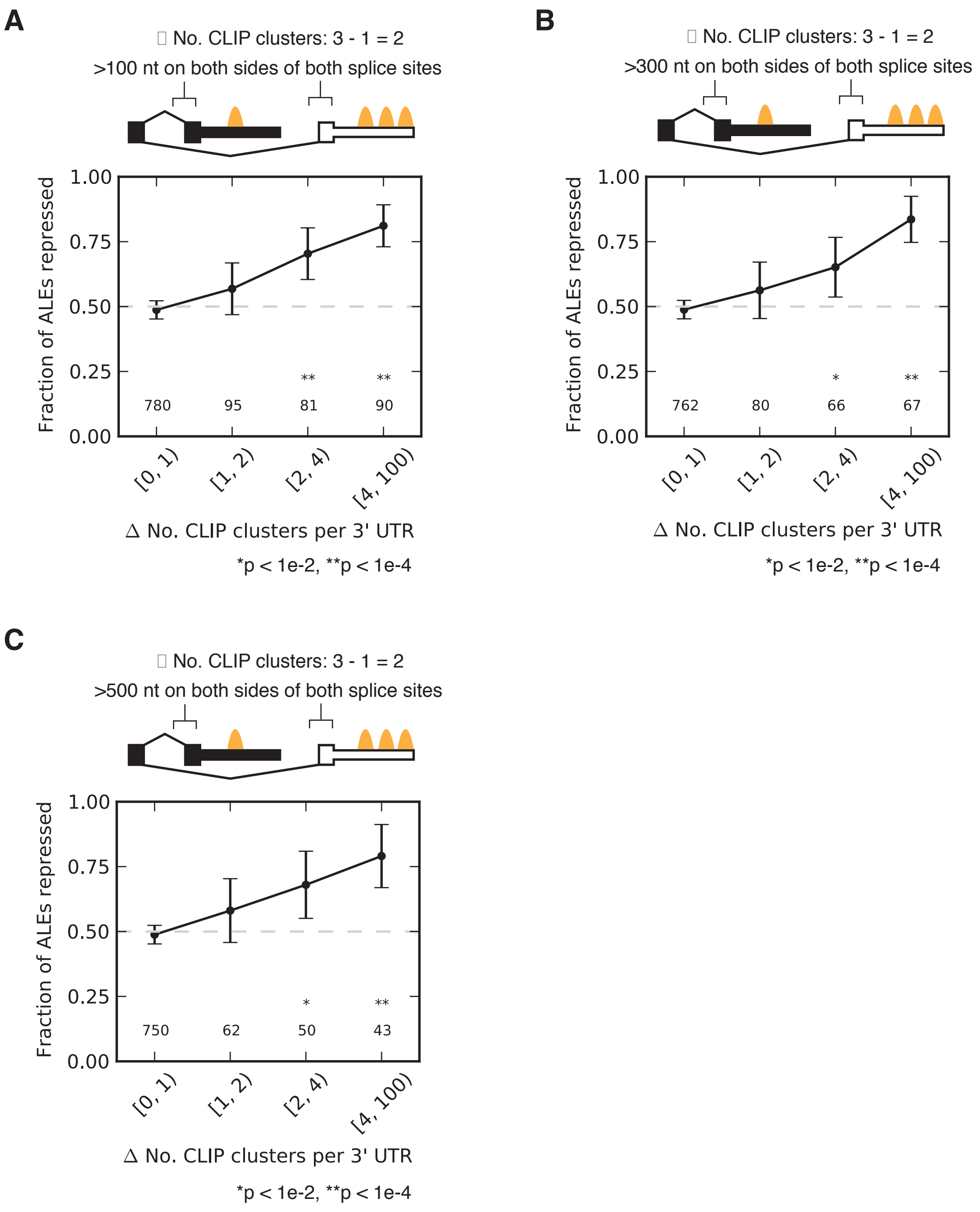

Figure S6. Alternative last exon abundance as a function of difference in number of CELF1 CLIP clusters between each 3' UTR. The fraction of ALEs that are significantly repressed following *CELF1* over-expression (MZ > 1.5), grouped by density in repressed 3' UTR minus density in enhanced 3' UTR. Analyses were performed using UTR pairs in which CELF1 binding is at least 100 nt (**A**), 300 nt (**B**), or 500 nt (**C**) away from both 3' splice sites of both alternative last exons.

A

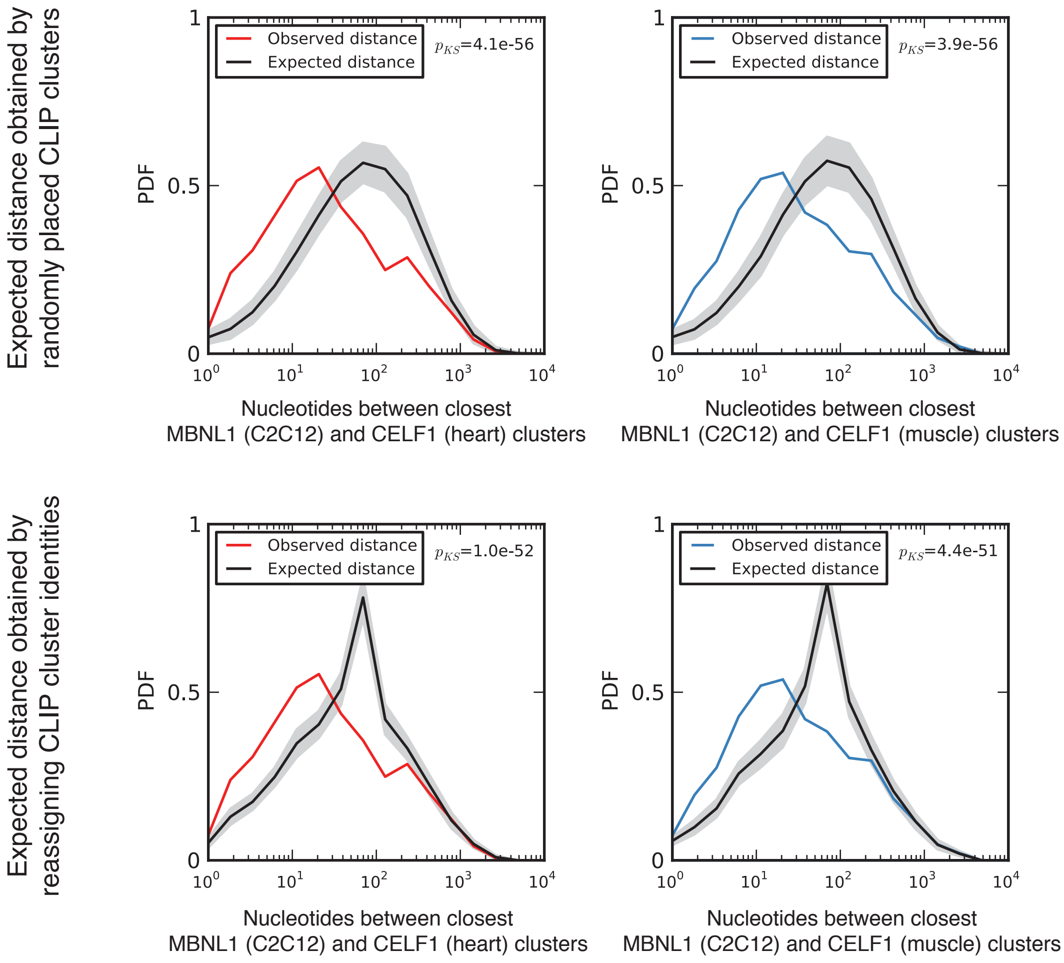

B

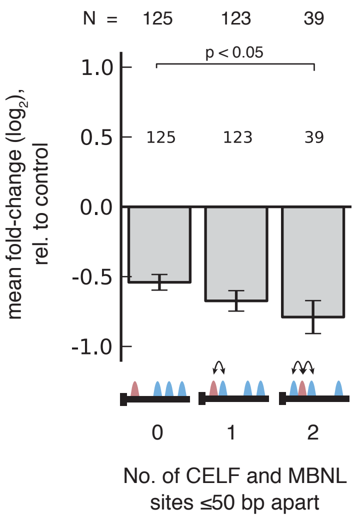

Figure S7. CELF1 and MBNL1 binding sites are closer than expected. A) The distribution of observed and expected distances between the centers of CELF1 and MBNL1 CLIP clusters. Plots on the left were generated using CELF1 CLIP clusters from heart, and on the right, CELF1 CLIP clusters from muscle. All MBNL1 CLIP clusters were derived from myoblasts. Expected distances for plots in the top row were computed by reassigning the clusters to random locations within 3' UTRs. For plots on the bottom, expected distances were computed by keeping the clusters in the same location, but randomly reassigning clusters as being bound by CELF1 or MBNL1. B) Expression change following CELF1 induction in muscle (7 d versus control) for genes with 1 MBNL1 CLIP cluster and more than 1 CELF1 CLIP cluster, grouped by the number of CELF1 sites < 50 nt away from the MBNL1 site and normalized to genes with no CELF1 sites < 50 nt away from the Mbnl1 site.

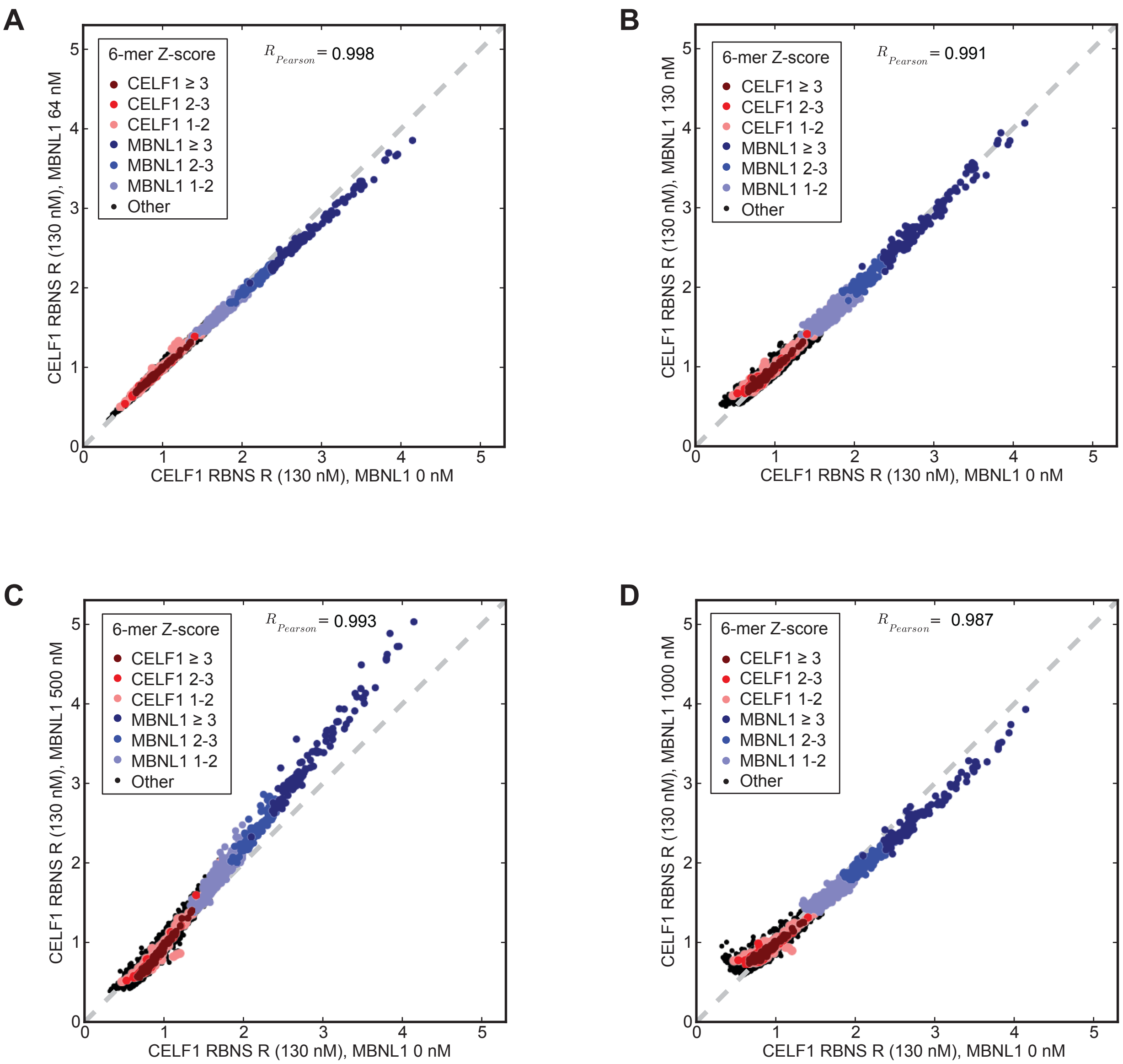

Figure S8. MBNL1 presence does not affect in vitro binding of CELF1 to RNA. 130 nM tagged CELF1 and various concentrations of MBNL1 were equilibrated in vitro with random RNA 40mers in an RNA Bind-n-Seq experiment. The tagged CELF1 was pulled-down and bound 40mers were eluted and sequenced. RBNS R was calculated as the frequency of the 6mer in the pulldown/input libraries, and all 6mers were classified as weak, medium, or strong (RBNS R Z-score of 1-2, 2-3, or ≥3, respectively) CELF1 or MBNL1 6mers, or none of the above ("Other") using the data from Lambert et al. 2014. The x-axis of all panels shows RBNS R values of 130 nM CELF1 incubated with no MBNL1, and the y-axis shows RNBS R values of 130 CELF1 incubated with the following concentrations of MBNL1: 64 nM (**A**), 130 nM (**B**), 250 nM (main text Fig. 5F), 500 nM (**C**), or 1000 nM (**D**).
